# Mental health and family functioning among physically healthy Syrian refugees resettled in Ontario, Canada post 2015: A cross-sectional study

**DOI:** 10.1371/journal.pone.0354919

**Published:** 2026-08-27

**Authors:** Bahia Abdallah, Abir Abdel Rahman, Safoura Zangiabadi, Hala Tamim

**Affiliations:** 1 Alice Ramez Chaghoury School of Nursing, Lebanese American University, Beirut, Lebanon; 2 Faculty of Health Sciences, University of Balamand, Balamand, Lebanon; 3 Department of Kinesiology and Health Science, York University, Toronto, Ontario, Canada; American University of Madaba, JORDAN

## Abstract

Refugees are often exposed to traumatic experiences before, during, and after migration, placing them at increased risk of mental health problems, including depression, anxiety, and post-traumatic stress disorder (PTSD). This cross-sectional study investigates the relationship between mental health and family functioning among Syrian refugees who resettled in Ontario, Canada, post-2015. The final sample included 398 parents residing in Ontario who reported good to excellent physical health. Validated tools, including the Depression Anxiety Stress Scales (DASS-21) and the Primary Care–posttraumatic stress disorder (PC-PTSD) scale for PTSD, were employed to assess PTSD, stress, anxiety, and depression. The General Functioning subscale of the McMaster Family Assessment Device was utilized to evaluate family functioning. The study indicated significant positive associations between poor family functioning and elevated levels of anxiety, stress, and PTSD, but no significant association with depression. In addition, lower socioeconomic status was linked to higher levels of depression, anxiety, and stress. Although gender differences were not statistically significant after adjustment, there was a trend toward fathers reporting poorer anxiety outcomes than mothers. The findings suggest that family functioning and socioeconomic factors are important factors associated with mental health outcomes among Syrian refugee parents. Future longitudinal research is needed to further examine these relationships and enhance understanding of the factors influencing mental health in refugee populations.

## Introduction

According to the UNHCR Global Trends: Forced Displacement in 2023 report, by the end of 2023 an estimated 117.3 million people worldwide were forcibly displaced as a result of persecution, conflict, violence, human rights violations, or public disorder [1]. The total forcibly displaced population has more than doubled in the last ten years, underscoring the accelerating scale and persistence of forced migration worldwide [1]. Two thirds of these displaced come from Syria, Ukraine, Afghanistan, Myanmar, and Somalia. Since the Syrian conflict began, over 250,000 Syrians have died, and more than 11 million have fled the country, many heading to Lebanon, Jordan, Turkey, the US, or Canada [2,3].

Refugees often experience extreme trauma before, during, and after migration, like witnessing violence or losing family, torture, or sexual violence [3]. Systematic reviews show high prevalence of depression, anxiety, and post-traumatic stress disorder (PTSD) among refugee populations in both acute humanitarian settings and post-resettlement contexts [4]. War-affected populations have significantly higher rates of mental disorders than people who have not lived through conflict [5]. Recent meta-analyses estimate PTSD rates around 31.5%, depression around 49–50%, and anxiety similarly high in displaced populations [6,7]. Studies in Europe show PTSD rates among Syrians ranging from 26% to 45%, depression from 40% to 45%, and anxiety from 30% to 32% [7].

Mental health is a major concern among refugee populations because forced migration is often accompanied by exposure to traumatic events and prolonged stressors that can adversely affect psychological well-being. Refugees may experience violence, persecution, loss of loved ones, displacement, and disruption of social networks before and during migration, followed by challenges such as economic hardship, social isolation, language barriers, and uncertainty during resettlement [8–11]. These trauma- and displacement-related stressors have been consistently associated with poor mental health outcomes, including depression, anxiety, stress, and post-traumatic stress disorder (PTSD) [8–10]. Given the high burden of mental health problems among refugee populations, understanding the factors associated with mental health outcomes is essential to inform research, policy, and support services for refugees and their families [8–10].

Data gathered from studies done in Sweden and Germany showed a prevalence of PTSD, depression, and anxiety as the key issues, ranging from 20.5 to 35.7%, from 20 to 43.9%, and from 19.3 to 31.8%, respectively [12,13]. Another extensive meta-analysis of child and adolescent refugees resettled in Europe and Malaysia provided a prevalence of the same major issues of PTSD, depression, and anxiety (averaging 22.7%, 13.8%, and 15.8%, respectively). Further to this, they also reported encouraging lower levels of PTSD and depression after 2-plus years of residence [12].

Forced migration adds a number of stresses to the trauma associated with armed conflict, which can worsen mental health status. These stressors include family separation, fewer prospects for employment, insecure access to public services, and uncertainty about the future [14]. Family functioning (communication, emotional connection, and coping) has a large influence on refugee mental health. Poor family functioning—such as worry over family back home, family separation, and loneliness—correlates with worse mental health [14,15]. Conversely, strong family cohesion and stable family relationships support the family’s role as a buffer against mental health issues [16]. Among refugee youth, family functioning is predictive of mental health outcomes and resilience. Family support can provide stability and security, enabling members to rely on each other to cope with the new experience and past traumas, or the trauma and excessive stress can hinder an individual’s ability to cope, negatively affecting other family members, depending on the individual [16,17].

Evidence from refugee youth in Australia, Canada, and the United States indicates that strong family functioning correlates with reduced depression, anxiety, and behavioral problems [5].

Because trauma exposure and displacement stressors accumulate, mental health among refugees often worsens over time. Longitudinal studies show depression and anxiety increasing in the first 1–3 years post-resettlement [18]. The cumulative impact of trauma and displacement often worsens over time, with longitudinal studies showing rising rates of depression and anxiety in the first years after resettlement [4]. In addition, an increasing amount of research is being done on the reciprocal effects that trauma can have on other family members’ psychological well-being and the generational effects that can last for a very long time [19]. Children who were refugees and whose parents had experienced significant trauma reported higher rates of common mental disorders than children whose parents had not reported significant trauma [20]. They also found that low parental engagement, poor family communication, and child abuse served as important mechanisms through which this trauma was transmitted. Previous research in this field has looked at underlying biological, societal, and economic reasons that could be responsible for the unfavorable mental health outcomes observed among children of war survivors, which can last into adulthood [21]. However, the family can also serve as a vital support system and means of overcoming the pressures and misfortune, including the challenges that come with uprooting and resettling refugee families [22].

The family plays a central role in helping refugees cope with the hardships of migration by fostering unity, shared meaning, and continuity of legacy [23]. Research shows that a family systems approach is essential, as war and forced displacement create vulnerabilities at the family level but also highlight the protective role of family in supporting individual recovery from trauma [24]. Refugee mental health is strongly shaped by family functioning, and there is growing recognition that interventions should move beyond an individual focus to include family- and community-based programs grounded in evidence-based practices [25].

Mental health research among Arab immigrants and refugees in Canada, including Syrian refugees, is scarce, leaving major gaps in understanding their unique stressors and support systems. This limited evidence base highlights the importance of the current research in providing culturally grounded insights that can inform responsive and equitable mental health care.

This study aims to explore the relationship between family functioning, socio-demographic, migration-related, and health-related factors with mental health outcomes, especially in the case of Syrian refugees in Canada.

## Methodology

### Study participants

This was a cross-sectional study of 540 Syrian refugee parents interviewed between March 3, 2021, and March 10, 2022. Out of whom 398 participants who self-rated their physical health as good, very good, or excellent were included in the analysis to reduce the potential influence of physical health conditions on both mental health outcomes and family functioning, thereby allowing for a more focused assessment of the association between family functioning and mental health. The inclusion criteria required participants to be Syrian refugee parents who resided in Ontario, had at least one child under 18, and resettled in the country after 2015. Participants were recruited via convenience sampling, facilitated by partnerships with community organizations such as Access Alliance Multicultural Health and the Arab Community Centre of Toronto. Recruitment was conducted through outreach activities and community contacts, whereby these organizations identified and shared contact information for Syrian families they had previously engaged and who had expressed interest in learning more about the study. Research assistants then contacted these individuals, explained the study in detail, and assessed their interest in participating. Surveys were administered by trained research assistants fluent in Arabic (Syrian dialect), and all responses were collected using Qualtrics, a secure, password-protected online platform. No identifying information was shared with community organizations, and participant confidentiality was strictly maintained.

### Ethical considerations

The study obtained approval from the York University Research Ethics Board (Certificate # e2019-128) and was conducted in accordance with the ethical standards of the Helsinki Declaration. The recruitment and interviews were conducted through telephone interviews to adhere to social distancing measures due to the COVID-19 pandemic. Hence, prior to the survey administration, research assistants emailed participants a digital copy of the consent form and explained the study’s objective. For those, without email, the consent form was explained verbally over the phone. Participants were also informed of the voluntary nature of their participation and their option to opt out at any time without any consequences. Individuals who chose not to participate or who withdrew from the study were not disadvantaged in any way and continued to have access to the same services and support provided through the participating community organizations. Research assistants then explained the consent form and addressed any questions or concerns raised by the participants. Subsequently, the research assistants recorded the audio of the participants’ oral consent. Participants were compensated with a $20 honorarium in recognition of their time and effort. Participants who reported poor self-rated mental health were referred to appropriate local mental health agencies for support, in accordance with the study protocol. Lastly, all survey responses were de-identified, with any identifying information stored separately and securely maintained on encrypted, password-protected servers accessible only to the research team.

### Measures and outcomes

The main outcome of the study was mental health, particularly levels of depression, anxiety, stress, and PTSD among Syrian refugee parents. For the assessment of mental health, the Arabic-translated version of the Depression Anxiety Stress Scales (DASS-21) was employed [18]. DASS-21 is a widely recognized self-report instrument consisting of 21 items. Developed as a condensed version of the original 42-item DASS, it measures three key mental health domains of stress, anxiety, and depression based on the respondents’ experiences over the preceding week. Each subscale consists of seven items, utilizing a 4-point Likert scale ranging from “Did not apply to me at all” to “Applied to me very much, or most of the time." To compute total scores for each subscale, individual item scores are multiplied by two to obtain scores ranging from 0 to 42, with higher scores indicating greater severity of symptoms. These scores are then assessed on a severity scale, categorized as follows: normal (0–9 for depression, 0–7 for anxiety, and 0–14 for stress), mild/moderate (10–20 for depression, 8–14 for anxiety, and 15–25 for stress), and severe/extremely severe (21+ for depression, 15+ for anxiety, and 26+ for stress) [19]. In the present sample, the DASS-21 subscales demonstrated good internal consistency (depression: α = 0.84; anxiety: α = 0.76; stress: α = 0.84).

The Primary Care – posttraumatic stress disorder (PC-PTSD), developed by Prins (2003) et al., is a self-report screening tool for PTSD within primary care settings [17]. This screening instrument consists of 4 items designed to assess experiences within the last month that were perceived as “frightening, horrible, or upsetting." Participants responded to these items in a Yes/No format, where “No” was scored as 0 and “Yes” as 1, with individual scores ranging from 0 to 4. An optimal cut-off score of 3 has been identified for both men and women, indicating the threshold beyond which individuals are more likely to exhibit symptoms consistent with PTSD.

The primary independent variable, family functioning, was examined based on the General Functioning 12-item subscale (GF-12) of the McMaster Family Assessment Device (FAD) [26]. The 12-item FAD-GF was utilized as a single index measure to assess individuals’ overall level of family functioning. The 12-item FAD-GF includes six items reflecting healthy family functioning and six items indicating unhealthy functioning. Participants rated each item on a 4-point Likert scale ranging from “Strongly agree” to “Strongly disagree." Reverse scoring is applied to six negatively worded items to ensure consistency in interpretation. The total score was computed by averaging all item scores, with scores ranging from 1 (indicating best functioning) to 4 (indicating worse functioning). Thus, a higher score indicated greater perceived issues in the family’s overall functioning by the member. The GF-12 demonstrated excellent internal consistency in the present sample (Cronbach’s α = 0.96).

Moreover, other socio-demographic-, migration-, and health-related factors were collected from the participants. The socio-demographic factors considered for the study included gender (being a mother/father, with the mother being the reference category), age, number of children, highest level of education (none-elementary/secondary high school-diploma/ university; with none-elementary being the reference category), perceived language proficiency in English or French measured by the question, “Please rate your current overall ability in English or French, whichever you are more comfortable with”, with the answers ranging from 1 representing “Excellent” to 5 representing “Not at all”, and self-perceived socioeconomic status measured by the question “In your current condition, here in Canada, would you say most people would categorize a household like yours as?” (measured in a 5-point Likert scale ranging from “lower income” to “upper income"). The migration-related characteristics included the type of sponsorship involved in the process of becoming a refugee (government-assisted refugee (GAR)/ privately sponsored refugee (PSR)/ and other; with GAR being the reference category, and the number of years spent in Canada. Health-related factors included information on smoking (yes/no, with no being the reference category) and alcohol consumption (yes/ no; with no being the reference category) behaviors.

### Statistical analyses

Descriptive statistics were used to summarize outcome variables, including mental health outcomes of depression, anxiety, stress, and PTSD. Means and standard deviations (SD) were reported for continuous variables, while frequencies and percentages were stated for categorical variables. Univariable and multivariable linear mixed-effects models were used to examine the associations between the independent variables and each mental health outcome (anxiety, depression, stress, and PTSD). All models included a random intercept for family identifier to account for clustering of participants within families. Unadjusted and adjusted unstandardized regression coefficients (B), standard errors (SE), and p-values were reported.

The proportion of missing data across study variables was minimal (<5%). Therefore, complete-case data were used, and listwise deletion was applied. All regression models were adjusted for Family ID to account for the clustering effect within families. All statistical analyses were conducted using the Statistical Package for the Social Sciences (SPSS), version 28.0. The statistical significance level was set at p < 0.05.

## Results

Table 1 displays the descriptive statistics summarizing the characteristics of the study participants, along with the bivariate relationship between family functioning, socio-demographic, migration, and health-related factors with mental health outcomes of depression, anxiety, stress, and PTSD. The majority were mothers (62.3%) and had a mean number of 3.20 children (SD = 1.44). The average age of respondents was 39.13 (SD = 7.17) with an average duration of 3.86 years spent in Canada (SD = 1.53). Furthermore, over half of the participants (52.8%) had a secondary or university-level education, while 61.1% reported being unemployed.

**Table 1:** Participant characteristics and bivariate associations with mental health outcomes among Syrian refugees reporting good physical health.

Factor			Depression		Anxiety		Stress		PTSD	
	Number (%)	Mean (SD)	B (SE)	p-value	B (SE)	p-value	B (SE)	p-value	B (SE)	p-value
**Family Functioning**		1.75 (0.48)	1.36 (0.73)	0.062	2.27 (0.55)	**<0.001**	2.47 (0.78)	**0.002**	0.63 (0.15)	**<0.001**
**Socio-demographic characteristics**										
**Gender**										
**Mother**	248 (62.3)		REF							
**Father**	150 (37.7)		−0.70 (0.55)	0.204	−0.88 (0.44)	**0.046**	−1.30 (0.62)	**0.038**	−0.10 (0.13)	0.449
**Age**		39.13 (7.17)	−0.069 (0.046)	0.132	−0.042 (0.035)	0.229	−0.10 (0.05)	**0.045**	−0.011 (0.01)	0.277
**Number of Children**		3.20 (1.44)	0.057 (0.25)	0.820	0.104 (0.19)	0.584	−0.061 (0.27)	0.820	0.101 (0.051)	**0.049**
**Education**										
**None/Elementary**	57 (14.3)		REF							
**Secondary/High School/Diploma**	210 (52.8)		0.40 (0.94)	0.673	0.17 (0.73)	0.819	0.87 (1.04)	0.403	0.13 (0.21)	0.531
**University**	131 (32.9)		0.14 (1.04)	0.891	0.40 (0.81)	0.620	1.20 (1.14)	0.293	0.005 (0.22)	0.982
Language proficiency in English or French*		2.84 (1.14)	0.11 (0.29)	0.694	0.39 (0.22)	0.085	-0.054 (0.32)	0.865	0.029 (0.063)	0.648
**Working status**										
**Yes**	155 (38.9)		0.92 (0.63)	0.144	0.42 (0.49)	0.398	0.40 (0.70)	0.563	0.11 (0.14)	0.428
**No**	243 (61.1)		REF							
Socioeconomic status**		2.21 (1.01)	−0.68 (0.33)	**0.039**	−0.60 (0.25)	**0.017**	−1.04 (0.36)	**0.004**	−0.14 (0.07)	**0.049**
**Migration related factors**										
**Sponsorship**										
**Governmental**	132 (33.2)		REF							
**Private**	252 (63.3)		−0.97 (0.78)	0.215	0.091 (0.60)	0.880	0.44 (0.84)	0.602	−0.30 (0.16)	0.064
**Other**	14 (3.5)		0.33 (1.98)	0.870	−1.41 (1.53)	0.359	0.07 (2.16)	0.973	−0.31 (0.42)	0.451
**Number of years in Canada**		3.86 (1.53)	−0.143 (0.23)	0.532	−0.088 (0.18)	0.620	0.044 (0.25)	0.862	−0.006 (0.049)	0.906
**Health-related Factors**										
**Alcohol (Yes)**	63 (15.8)		−0.35 (0.90)	0.702	−0.73 (0.70)	0.297	−0.45 (0.99)	0.651	−0.05 (0.20)	0.808
**Smoking (Yes)**	72 (18.1)		−0.15 (0.78)	0.848	0.14 (0.61)	0.823	−0.36 (0.87)	0.680	−0.01 (0.18)	0.970

*Scale from 1–5 (1 = Excellent and 5 = Poor).

**Scale form 1–5 (1 = Lower income and 5 = Upper income).

Note. B = unstandardized coefficient; SE = standard error; REF = Reference. All models are unadjusted.

From the original sample of 398 respondents, effective analytic sample sizes ranged from 386 to 389, indicating approximately 2–3% missing data per model. Variance inflation factors (VIFs) were examined for all predictors in each model, and none of the VIF values exceeded 4, suggesting that multicollinearity was not a concern. Residual normality was assessed as part of the regression diagnostics, and the assumptions for the regression analyses were considered to be adequately met.

The findings of the univariable linear mixed-effects regression analyses demonstrated positive associations between family functioning and anxiety (B = 2.27, *p* < 0.001), stress (B = 2.47, *p* = 0.002), and PTSD (B = 0.63, *p* < 0.001). Although family functioning was positively associated with depression (B = 1.36, *p* = 0.062), this association did not reach statistical significance.

Table 2 indicates the levels of depression, anxiety, stress, and PTSD among respondents. The majority of participants reported normal levels of depression (84.4%), anxiety (83.9%), and stress (91.7%). A small percentage, 2.8%, 2.5%, and 3.3%, respectively, reported severe to extremely severe levels of depression, anxiety, and stress with mean scores (SD) of 4.15 (6.45), 3.08 (4.96), and 5.97 (7.08). Additionally, 16.6% of individuals screened positive for symptoms indicative of PTSD, meeting the cut-off score of 3 or higher.

**Table 2:** Depression, anxiety, stress, and PTSD levels among Syrian refugees in Canada who self-rated their physical health as good, very good, or excellent.

	Mental Health Outcomes^a^							
	Depression		Anxiety		Stress		PTSD	
	N	%	N	%	N	%	N	%
**Normal**	336	84.4%	334	83.9%	365	91.7%		
**Mild/Moderate**	48	12.1%	53	13.3%	19	4.8%		
**Severe/Extremely Severe**	11	2.8%	10	2.5%	13	3.3%		
**Yes**							66	16.6%
Mean (SD)	4.15 (6.45)		3.08 (4.96)		5.97 (7.08)		0.80 (1.39)	

^a^Missing data: Depression (n = 3), Anxiety (n = 1), Stress (n = 1), PTSD (n = 0). Percentages were calculated using the total sample; they may not total 100% because of missing data and rounding.

The results of the multivariable linear mixed-effects models are presented in Table 3. After adjusting for all variables considered in the study, family functioning remained significantly associated with anxiety, stress, and PTSD. Participants with higher family functioning scores exhibited higher levels of anxiety (B = 1.56, *p* = 0.005), stress (B = 1.72, *p* = 0.035), and PTSD (B = 0.56, *p* < 0.001). Gender was not significantly associated with any of the mental health outcomes after adjustment, although fathers tended to report lower anxiety than mothers (B = −1.13, *p* = 0.053). The results also demonstrated a significant association between socioeconomic status and depression, anxiety, and stress. Participants with lower perceived socioeconomic status reported higher levels of depression (B = −0.74, *p* = 0.036), anxiety (B = −0.62, *p* = 0.019), and stress (B = −1.28, *p* < 0.001).

**Table 3:** Multivariable linear regression analysis of factors associated with stress, anxiety, depression, and PTSD among Syrian refugees reporting good physical health.

Factor	Depression Adjusted B (SE)	p	Anxiety Adjusted B (SE)	p	Stress Adjusted B (SE)	p	PTSD Adjusted B (SE)	p
**Family Functioning**	0.78 (0.75)	0.298	1.56 (0.55)	**0.005**	1.72 (0.82)	**0.035**	0.56 (0.16)	**<0.001**
**Socio-demographic characteristics**								
**Gender**								
**Mother**	REF		REF		REF		REF	
**Father**	−0.53 (0.74)	0.480	−1.13 (0.58)	0.053	−1.22 (0.82)	0.139	−0.02 (0.17)	0.929
**Age**	−0.05 (0.06)	0.373	−0.04 (0.04)	0.304	−0.12 (0.06)	**0.046**	−0.02 (0.01)	0.176
**Number of Children**	−0.13 (0.32)	0.679	−0.06 (0.24)	0.803	0.11 (0.35)	0.749	0.10 (0.07)	0.134
**Education**								
**None/Elementary**	REF		REF		REF		REF	
**Secondary/High School/Diploma**	−0.21 (1.04)	0.841	0.01 (0.81)	0.993	−0.23 (1.16)	0.840	−0.17 (0.23)	0.476
**University**	−0.45 (1.27)	0.725	−0.87 (0.98)	0.375	−0.98 (1.41)	0.489	−0.19 (0.28)	0.497
**Language proficiency**	−0.09 (0.37)	0.799	0.51 (0.28)	0.072	0.07 (0.41)	0.862	−0.05 (0.08)	0.542
**Working status (Yes)**	0.28 (0.75)	0.704	−0.28 (0.58)	0.634	−0.68 (0.83)	0.414	−0.03 (0.17)	0.859
**Socioeconomic status**	−0.74 (0.35)	**0.036**	−0.62 (0.26)	**0.019**	−1.28 (0.38)	**<0.001**	−0.13 (0.08)	0.098
**Migration-related factors**								
**Sponsorship**								
**Government**	REF		REF		REF		REF	
**Private**	0.73 (0.97)	0.457	−0.88 (0.72)	0.218	−1.25 (1.06)	0.238	0.13 (0.21)	0.529
**Other**	0.63 (1.96)	0.748	0.77 (1.45)	0.596	−0.81 (2.14)	0.706	0.00 (0.42)	0.994
**Years in Canada**	0.03 (0.25)	0.923	0.27 (0.19)	0.154	0.27 (0.28)	0.334	0.02 (0.05)	0.704
**Health-related Factors**								
**Alcohol (Yes)**	0.43 (0.95)	0.652	−0.35 (0.71)	0.625	0.24 (1.04)	0.819	0.19 (0.21)	0.370
**Smoking (Yes)**	0.38 (0.86)	0.655	0.88 (0.66)	0.187	0.54 (0.95)	0.568	0.01 (0.19)	0.961

*Scale from 1–5 (1 = Excellent and 5 = Poor).

**Scale form 1–5 (1 = Lower income and 5 = Upper income).

Note. B = Adjusted unstandardized coefficient; SE = standard error; REF = Reference. All coefficients are from multivariable-adjusted models.

## Discussion

Our study found relatively low levels of severe psychological distress among Syrian refugee parents in Canada, with only 2.8%, 2.5%, and 3.3% reporting severe to extremely severe depression, anxiety, and stress, respectively. These findings differ substantially from previous studies reporting high rates of mental health disorders among refugee populations. Extant research has documented depression prevalence ranging from 28% to 54%, PTSD from 9% to 62%, and anxiety rates reaching 64% among Iraqi and Syrian refugees and other Arab refugee populations living in Western countries [2]. Similarly, studies conducted among Syrian refugees in Turkey reported prevalence rates of 36.1% for anxiety and 34.7% for depression, while a recent systematic review found pooled prevalence estimates of 40% for anxiety, 31% for depression, and 31% for PTSD among Syrian refugees resettled in high-income countries [17].

Several factors may explain these differences. Mental health outcomes among refugees are influenced not only by exposure to war, displacement, and trauma but also by post-migration conditions, including social support, family functioning, economic stability, access to healthcare, and opportunities for social integration [10,27]. Our findings support this perspective and suggest that refugee mental health cannot be understood solely through a trauma-focused lens. Rather, psychological well-being appears to be shaped by the broader social and environmental context in which resettlement occurs. A national Canadian study reported that many refugees experienced positive mental health, with a stronger sense of belonging, greater social connectedness, and lower levels of perceived discrimination associated with better mental health outcomes [28]. Similar findings have been reported in Australia, where resettled refugees demonstrated declining psychological distress and improved social integration over time, suggesting substantial recovery following successful resettlement [29].

Canada’s comprehensive refugee support infrastructure may partly contribute to these favorable outcomes. Refugees arriving through Government-Assisted Refugee, Privately Sponsored Refugee, and Blended Visa Office-Referred programs receive orientation services, housing assistance, language training, educational and employment support, settlement services, financial assistance, and access to healthcare. These resources may facilitate social integration, reduce post-migration stressors, and strengthen resilience during resettlement. Such findings reinforce growing evidence that host-country support systems can play a critical role in promoting mental health among refugee populations [11].

The present study also identified moderate levels of family dysfunction among Syrian refugee families. Although family functioning scores were somewhat poorer than those reported among Syrian forced migrants living in Sweden, differences may reflect variations in cultural adaptation processes, family composition, duration of resettlement, and available support services across countries [11,30]. More importantly, family functioning emerged as a significant determinant of anxiety, stress, and PTSD symptoms. Refugees who reported greater family dysfunction experienced poorer mental health outcomes, supporting previous research demonstrating the protective role of healthy family relationships during forced migration and resettlement [11,31]. Family systems often provide emotional support, foster a sense of security and belonging, facilitate communication and problem-solving, and assist family members in navigating unfamiliar environments and services [32].

An important finding of this study was the absence of an independent association between family functioning and depression after adjustment for other variables. Although family functioning was associated with depression in the bivariate analysis, this relationship did not remain significant in the multivariable model. Instead, socioeconomic status emerged as the primary predictor of depressive symptoms. This finding differs from studies that have identified family functioning as a significant determinant of depression among refugee populations. For example, Darwiche et al. (2023) [33] found that family functioning was associated with mental health outcomes among Syrian forced migrants, emphasizing the protective role of family relationships during resettlement.

The lack of a significant association between family functioning and depression in our study may reflect the multifactorial nature of depression among adult refugees. Depression is increasingly recognized as being influenced by a complex interaction of pre-migration trauma, ongoing socioeconomic hardship, perceived stress, social exclusion, and challenges related to adaptation and identity reconstruction [27]. Research among Syrian refugees in Canada has shown that depressive symptoms are strongly associated with perceived stress and perceived control during the resettlement process, while studies among Syrian refugee women have highlighted the influence of sociocultural and contextual factors, including trauma-related experiences and perceived autonomy. These findings suggest that depression may be more closely linked to individual psychosocial adaptation and structural determinants than to family functioning alone [28].

Our findings further support this interpretation, as lower socioeconomic status was significantly associated with higher levels of depression, anxiety, and stress. This is consistent with extensive evidence demonstrating that financial insecurity, unemployment, housing instability, and limited access to resources negatively affect mental health among refugee populations. Socioeconomic disadvantage may reduce parents’ ability to meet family needs, contribute to feelings of inadequacy and loss of control, and increase chronic stress, thereby affecting both parental and family well-being. The strong influence of socioeconomic status observed in this study highlights the importance of addressing structural determinants of health alongside psychosocial factors when designing refugee support programs [28].

Gender differences were less pronounced after adjustment for potential confounders. Although fathers tended to report lower anxiety levels than mothers, this association was only borderline significant. This finding is consistent with previous literature suggesting that refugee women may be more vulnerable to adverse mental health outcomes due to caregiving responsibilities, financial pressures, social isolation, gender-related stressors, and greater exposure to traumatic experiences [28,34].A scoping review of Arabic-speaking immigrants and refugees identified financial strain, social isolation, acculturation stress, and family role changes as major contributors to poor mental health, while social support, community belonging, and family cohesion acted as protective factors [34]. Our findings provide empirical support for these mechanisms by demonstrating that both family functioning and socioeconomic conditions are closely linked to mental health outcomes among Syrian refugee families.

Taken together, these findings suggest that refugee mental health is shaped by a complex interplay of family, socioeconomic, gender, and post-migration factors. While family functioning appears to protect against anxiety, stress, and PTSD, depression may be more strongly influenced by socioeconomic disadvantage and broader psychosocial adaptation processes. These findings highlight the need for comprehensive interventions that extend beyond trauma-focused approaches. Programs should incorporate family-strengthening strategies, mental health support services, socioeconomic assistance, employment and educational opportunities, and targeted support for refugee mothers. Strengthening social integration and community connectedness may further enhance psychological well-being and resilience among refugee families [28]. Future longitudinal studies are needed to clarify the pathways through which family functioning, socioeconomic conditions, and post-migration experiences interact to influence mental health over time and to identify modifiable protective factors that can inform policy and practice.

### Strengths and limitations

This study has several strengths. Examining both individual mental health and family functioning provides a more holistic view of adaptation and well-being after resettlement [27]. Targeting refugees who arrived post-2015 ensures the findings reflect the experiences of recent cohorts under current Canadian immigration and integration policies [35]. Conducting the research in Canada offers insight into how social supports, policy structures, and multicultural contexts shape outcomes. The use of validated mental health and family assessment tools strengthens the reliability of the findings and their applicability to clinical and community interventions. However, the Family Assessment Device (FAD) may not fully capture the complexity of family dynamics in refugee populations. Using a more culturally appropriate tool could help provide a more accurate representation of family functioning.

This study’s cross-sectional design and the use of convenience sampling limit our ability to infer causality and generalize these findings to all Syrian refugee parents in Canada. The findings should be interpreted with caution, and future research should employ longitudinal designs and consider using stratified random sampling to better understand the causal relationships and enhance representativeness. The measurements and outcomes were self-reported, subjecting the results to bias. Additionally, data collection occurred during the COVID-19 pandemic, which may have influenced participants’ mental health experiences, and thereby this context should be considered when interpreting the results.

## Conclusion

This cross-sectional study found that poorer family functioning was associated with elevated levels of anxiety, stress, and PTSD among physically healthy Syrian refugee parents resettled in Ontario. However, socioeconomic status was associated with higher levels of depression, anxiety, and stress. In addition, mothers reported poorer mental health outcomes than fathers. These findings highlight the importance of considering family functioning, socioeconomic circumstances, and gender when examining mental health among refugee populations. Given the cross-sectional nature of the study, causal relationships cannot be inferred. Further longitudinal studies are needed to better understand the temporal relationships between these factors and mental health outcomes and to inform the development of appropriate support strategies for refugee families.

## Supporting information

S1 File Questionnaire. (DOCX)

## References

[1] Global Trends report 2023; 2025. Available from: https://www.unhcr.org/media/global-trends-report-2023

[2] El-Refaay SM, Kenny C, Weiss S. Depression and anxiety among arab individuals in the United States: a meta-analysis. J Immigr Minor Health. 2025;27(2):329–50. doi: 10.1007/s10903-024-01648-9 39602001 PMC11903594

[3] Elshahat S, Moffat T, Iqbal BK, Newbold KB, Morshed M, Alkhawaldeh H, et al. “I thought we would be cherished and safe here”: understanding the multi-faceted nature of mental health among Arab immigrants/refugees in Ontario, Canada- the CAN-HEAL study. Soc Psychiatry Psychiatr Epidemiol. 2025;60(1):163–79. doi: 10.1007/s00127-024-02668-4 38664285

[4] Handiso DW, Paul E, Boyle JA, Shawyer F, Belsti Y, Enticott JC. Course of depression and anxiety among refugees and asylum seekers: a systematic review and meta-analysis of longitudinal studies. Int J Ment Health Addict. 2024;24(1):221–39. doi: 10.1007/s11469-024-01406-w

[5] Lerias D, Ziaian T, Arthur N, Augoustinos M, Pir T, Miller E. The mental health of refugee and migrant youth after settlement: outcomes of a multinational study. Community Ment Health J. 2025;61(7):1334–67. doi: 10.1007/s10597-025-01474-9 40465158 PMC12408779

[6] Baumgartner JS, Renner A, Wochele-Thoma T, Wehle P, Barbui C, Purgato M, et al. Impairments in psychological functioning in refugees and asylum seekers. Front Psychol. 2024;14:1295031. doi: 10.3389/fpsyg.2023.1295031 38259575 PMC10801113

[7] Ojha S, Thapa S, Thapa SB. Mental health problems among Syrian refugees in Nordic countries: a systematic review. Nord J Psychiatry. 2024;78(7):561–9. doi: 10.1080/08039488.2024.2403600 39282824

[8] Blackmore R, et al. Systematic review and meta-analysis: the prevalence of mental illness in child and adolescent refugees and asylum seekers. J Am Acad Child Adolesc Psychiatry. 2020;59(6):705–14. 31778780 10.1016/j.jaac.2019.11.011

[9] Bogic M, Njoku A, Priebe S. Long-term mental health of war-refugees: a systematic literature review. BMC Int Health Hum Rights. 2015;15:29. doi: 10.1186/s12914-015-0064-9 26510473 PMC4624599

[10] Porter M, Haslam N. Predisplacement and postdisplacement factors associated with mental health of refugees and internally displaced persons: a meta-analysis. JAMA. 2005;294(5):602–12. doi: 10.1001/jama.294.5.602 16077055

[11] Gleeson C, Frost R, Sherwood L, Shevlin M, Hyland P, Halpin R, et al. Post-migration factors and mental health outcomes in asylum-seeking and refugee populations: a systematic review. Eur J Psychotraumatol. 2020;11(1):1793567. doi: 10.1080/20008198.2020.1793567 33312450 PMC7717836

[12] Blackmore R, Boyle JA, Fazel M, Ranasinha S, Gray KM, Fitzgerald G, et al. The prevalence of mental illness in refugees and asylum seekers: a systematic review and meta-analysis. PLoS Med. 2020;17(9):e1003337. doi: 10.1371/journal.pmed.1003337 32956381 PMC7505461

[13] Georgiadou E, et al. Prevalence of mental distress among Syrian refugees with residence permission in Germany: a registry-based study. Front Psychiatry. 2018;9:393. 30210373 10.3389/fpsyt.2018.00393 PMC6121182

[14] Nguyen TP, Al Asaad M, Sena M, Slewa-Younan S. Loneliness and social isolation amongst refugees resettled in high-income countries: a systematic review. Soc Sci Med. 2024;360:117340. doi: 10.1016/j.socscimed.2024.117340 39293283

[15] Belau MH, Becher H, Kraemer A. Impact of family separation on subjective time pressure and mental health in refugees from the Middle East and Africa resettled in North Rhine-Westphalia, Germany: a cross-sectional study. Int J Environ Res Public Health. 2021;18(21):11722. 34770236 10.3390/ijerph182111722 PMC8582773

[16] Hadfield K, Al-Soleiti M, Dajani R, Mareschal I, Panter-Brick C. Father involvement and child development: a prospective study of syrian refugee families. J Child Fam Stud. 2024;33(3):1029–42. doi: 10.1007/s10826-024-02809-y

[17] Acarturk C, McGrath M, Roberts B, Ilkkursun Z, Cuijpers P, Sijbrandij M, et al. Prevalence and predictors of common mental disorders among Syrian refugees in Istanbul, Turkey: a cross-sectional study. Soc Psychiatry Psychiatr Epidemiol. 2021;56(3):475–84. doi: 10.1007/s00127-020-01941-6 32789561

[18] Prins A, Ouimette P, Kimerling R, Camerond RP, Hugelshofer DS, Shaw-Hegwer J, et al. The primary care PTSD screen (PC–PTSD): development and operating characteristics. Prim Care Psychiatry. 2004;9(1):9–14. doi: 10.1185/135525703125002360

[19] Lovibond PF, Lovibond SH. The structure of negative emotional states: comparison of the Depression Anxiety Stress Scales (DASS) with the Beck Depression and Anxiety Inventories. Behav Res Ther. 1995;33(3):335–43. doi: 10.1016/0005-7967(94)00075-u 7726811

[20] Mak C, Wieling E. A systematic review of evidence-based family interventions for trauma-affected refugees. Int J Environ Res Public Health. 2022;19(15):9361. doi: 10.3390/ijerph19159361 35954717 PMC9367780

[21] Patterson JE, Abu-Hassan HH, Vakili S, King A. Family focused care for refugees and displaced populations: global opportunities for family therapists. J Marital Fam Ther. 2018;44(2):193–205. doi: 10.1111/jmft.12295 29194696

[22] Gangamma R. A Phenomenological study of family experiences of resettled Iraqi refugees. J Marital Fam Ther. 2018;44(2):323–35. doi: 10.1111/jmft.12251 28677838

[23] Xiong ZB, Her M, Kira M, Belgrade AJ, Pattipati MA, Xiong GN, et al. Role of family in refugee adjustment: experiences of Hmong, Somali, and Syrian refugees in the USA. Adv Resil Sci. 2021;2(4):285–301. doi: 10.1007/s42844-021-00043-9

[24] Devakumar D, Birch M, Osrin D, Sondorp E, Wells JCK. The intergenerational effects of war on the health of children. BMC Med. 2014;12:57. doi: 10.1186/1741-7015-12-57 24694212 PMC3997818

[25] Flanagan N, et al. Crossing borders: a systematic review of risk and protective factors for the intergenerational transmission of trauma in refugee and asylum-seeking families. Eur J Psychotraumatol. 2020. 10.1080/20008198.2020.1790283 PMC7534369 33062205

[26] Boterhoven de Haan KL, Hafekost J, Lawrence D, Sawyer MG, Zubrick SR. Reliability and validity of a short version of the general functioning subscale of the McMaster Family Assessment Device. Fam Process. 2015;54(1):116–23. doi: 10.1111/famp.12113 25385473

[27] Miller KE, Rasmussen A. The mental health of civilians displaced by armed conflict: an ecological model of refugee distress. Epidemiol Psychiatr Sci. 2017;26(2):129–38. doi: 10.1017/S2045796016000172 27040595 PMC6998767

[28] Beiser M, Hou F. Predictors of positive mental health among refugees: results from Canada’s General Social Survey. Transcult Psychiatry. 2017;54(5–6):675–95. doi: 10.1177/1363461517724985 28854860

[29] Nguyen TP, Slewa-Younan S, Rioseco P. Trajectories of psychological distress and social integration in newly resettled refugees: findings from the Building a New Life in Australia longitudinal study. Soc Psychiatry Psychiatr Epidemiol. 2024;59(8):1425–35. doi: 10.1007/s00127-023-02528-7 37393205 PMC11291576

[30] Siriwardhana C, Ali SS, Roberts B, Stewart R. A systematic review of resilience and mental health outcomes of conflict-driven adult forced migrants. Confl Health. 2014;8:13. doi: 10.1186/1752-1505-8-13 25177360 PMC4149800

[31] Sim A, Bowes L, Gardner F. The promotive effects of social support for parental resilience in a refugee context: a cross-sectional study with Syrian mothers in Lebanon. Prev Sci. 2019;20(5):674–83. doi: 10.1007/s11121-019-0983-0 30684214 PMC6541567

[32] Walsh F. Family resilience: a developmental systems framework. Eur J Dev Psychol. 2016;13(3):313–24. doi: 10.1080/17405629.2016.1154035

[33] Darwiche J, El Ghaziri N, Blaser J, Spini D, Suris J-C, Antonietti J-P, et al. Importance of asylum status, support programmes, and family unit functioning on the mental health of Syrian forced migrants in Switzerland: a longitudinal study. J Refug Stud. 2023;36(3):507–33. doi: 10.1093/jrs/fead032

[34] Turrini G, Purgato M, Ballette F, Nosè M, Ostuzzi G, Barbui C. Common mental disorders in asylum seekers and refugees: umbrella review of prevalence and intervention studies. Int J Ment Health Syst. 2017;11:51. doi: 10.1186/s13033-017-0156-0 28855963 PMC5571637

[35] Kaida L, Hou F, Stick M. The long-term economic integration of resettled refugees in Canada: a comparison of privately sponsored refugees and government-assisted refugees. J Ethn Migr Stud. 2019;46(9):1687–708. doi: 10.1080/1369183x.2019.1623017

