# Supplementary material for: Mental health and family functioning among physically healthy Syrian refugees resettled in Ontario, Canada post 2015: A cross-sectional study
Source: PLoS One. 2026 Aug 27;21(8):e0354919. doi: 10.1371/journal.pone.0354919 (PMC13521349; doi:10.1371/journal.pone.0354919)
Supplement: Supplementary file 1 [file pone.0354919.s001.docx]

**Questionnaire (2021)**

[1.	Participant ID:	3](#_Toc64986624)

[2.	Family ID Number:	3](#_Toc64986625)

[3.	Socio-Demographic Cluster	3](#_Toc64986626)

[20.	Integration Cluster	5](#_Toc64986627)

[32.	Social Support Cluster:	9](#_Toc64986628)

[38.	Housing Cluster	10](#_Toc64986629)

[44.	Employment and Income Cluster	11](#_Toc64986630)

[54.	Education Cluster	14](#_Toc64986631)

[Language Classes Cluster	15](#_Toc64986632)

[65.	Subjective Cluster	17](#_Toc64986633)

[Perceived Security Cluster	21](#_Toc64986634)

[Attitudes towards Schools Cluster:	22](#_Toc64986635)

[88.	Family Functioning Cluster	23](#_Toc64986636)

[101.	Family Roles Cluster (FR):	25](#_Toc64986637)

[111.	General Health Cluster	27](#_Toc64986638)

[Fear of Coronavirus-19 Cluster:	29](#_Toc64986639)

[131.	Mental Health Cluster	30](#_Toc64986640)

[135.	PTSD Cluster:	32](#_Toc64986641)

[140.	DASS 21 Cluster:	33](#_Toc64986642)

[162.	Healthcare Services Cluster:	38](#_Toc64986643)

[169.	Oral Health Cluster:	39](#_Toc64986644)

[Final Questions Cluster:	40](#_Toc64986645)

[1.	Child Information Cluster	41](#_Toc64986646)

1. Participant ID:
2. Family ID Number:
3. Socio-Demographic Cluster:

The following set of questions will address general demographics.

معلومات ديموغرافية /شخصية: الأسئلة التالية تتناول المعلومات الشخصية بصفة عامة.

4. Is the participant the mother or father?

هل المشارك الأم او الاب؟

- Mother الأم /
- Father الاب /

5. What is your age?

ما هو عمرك؟

# 6. Are you ………………..? هل أنت ...................؟

# Single/unmarried/ أعزب- غير متزوج

# Married/ متزوج

# Common law/ علاقة قانونية

# Separated, but still legally married منفصل ولكن لا أزال متزوجاً من الناحية القانونية(

# Divorced/ مطلق

# Widowed/ أرمل

# Other/ غير ذلك

7. Are you and your spouse related?

هل يوجد قرابة بينك وبين زوجك/زوجتك؟

- First cousins / قرابة من درجة اولى
- Second cousins / قرابة من درجة ثانية
- Distant relatives / قرابة بعيدة
- No relation / لا قرابة
- Don’t know/prefer not to answer/ لا أعرف/ أفضل عدم القول

8. Which of the following best describes your racial or ethnic group?

أي مما يلي يصف بشكل أفضل المجموعة العرقية والاثنية التي تنتمي إليها؟

- Arab/ عربي
- Armenian/ أرميني
- Kurdish/ كردي
- Other/ غير ذلك

9. What language would you feel most comfortable speaking in with others?

ما هي اللغة التي تشعر فيها بالارتياح الشديد عند التحدث مع الآخرين؟

- Arabic/ العربية
- Armenian/ الأرمينية
- Kurdish/ الكردية
- Turkish/ التركية
- English/ الإنجليزية
- French/ الفرنسية
- Other/

10. How many children do you have? ___________

كم عدد الأطفال لديك ؟ ___________

11. What is the age of your oldest child?

كم عمر طفلك البكر؟

12. What is the age of your youngest child?

كم عمر طفلك الأصغر؟

13. Not counting your children or your spouse, how many other relatives (anyone you consider family) do you currently have living in Toronto?

دون حساب شريك حياتك أو أطفالك، كم عدد أقاربك الذين يعيشون حاليا في تورنتو؟

14. What is the highest level of formal education that you have completed?

ما هو أعلى مستوى دراسي أنهيته في التعليم الرسمي؟

- None/ لا يوجد
- Elementary school (up to grade 6)/ مدرسة ابتدائية (حتى الصف ٦)
- Secondary school (up to grade 9)/ مدرسة إعدادية (حتى الصف ٩)
- High school (up to grade 12)/ مدرسة ثانوية (حتى الصف ١٢)
- Trade certificate/diploma/ شهادة/دبلوم تجارة
- College diploma/ شهادة معهد
- University degree (BA/BSc)/ درجة جامعية (ليسانس آداب بكالوريوس علوم)
- Master degree/ درجة الماجستير
- PhD/ درجة الدكتوراه
- Professional degree (engineering, dentistry, law, etc.)

درجة جامعية عالية (هندسة، طب أسنان، قانون، إلخ)

15. What is your religion?

ما هي ديانتك؟

- Muslim/ مسلم
- Christian/ مسيحي
- Prefer not to answer/ أفضل عدم الإجابة
- Other/ غير ذلك

16. How religious do you consider yourself?

الى أي مدى تعتبر نفسك شخص متدين؟

- Not at all religious/ لست متديناً على الإطلاق
- A little religious/ متدين قليلاً
- Fairly religious/ متدين
- Very religious/ متدين جداً
- Other
- Do not know/ لا أعرف
- Prefer not to answer/ أفضل عدم الإجابة

17. What year did you leave Syria? ________

متى غادرت سوريا؟ __________

18. After displacement, in which country outside of Syria did you primarily live?

في أي دولة كنت تعيش خارج سوريا قبل المجيء إلى كندا؟

- Jordan/ الأردن
- Lebanon/	لبنان
- Turkey/ تركيا
- Egypt/ مصر
- Iraq/ العراق
- Other/ غير ذلك

19. During your refuge outside Syria, did you live in a refugee camp?

خلال فترة لجوئك خارج سوريا، هل عشت في مخيم للاجئين؟

- Yes/ نعم
- No/ لا
- Prefer not to answer/ أفضل عدم القول

20. When did you arrive to Canada? (month/year) _______

متى وصلت إلى كندا؟ (شهر/سنة) ___________

21. Integration Cluster :
22. Are you in Canada as a privately sponsored refugee, a refugee sponsored by the Canadian government or on a blended visa-office?

هل أنت في كندا:

- Government assisted refugee (GAR)/ لاجئ تحت كفالة الحكومة
- Privately sponsored refugee (PSR)/ لاجئ تحت كفالة خاصة
- Blended visa-office refugee (BVOR)/ لاجئ من خلال مكتب تأشيرات مختلط
- Unsure/ غير متأكد
- Other/ غير ذلك

23. Who is your private sponsor?

من هو كفيلك الخاص؟

- Family member(s)/ فرد (أفراد) عائلة
- Religious organization/ منظمة دينية
- Community organization/ منظمة مجتمعية
- A group of private individuals (e.g. Group of 5)

مجموعة من الأفراد للكفالة الخاصة (على سبيل المثال مجموعة من ٥)

- Other

24. If PSR or BVOR, how satisfied are you with the support and services you receive from your sponsor?

إذا كنت على كفالة خاصة او كفالة مختلطة، ما مدى رضاك عن الدعم والخدمات التي تتلقاها من كفيلك؟

- Very satisfied/ راض جداً
- Satisfied/ راضٍ
- Neither satisfied or unsatisfied/ لا راضي ولا غير راضي
- Unsatisfied/ غير راضي
- Very unsatisfied/ غير راضي بشدة

25. Are you a Canadian citizen?

هل انت مواطن كندي؟

- Yes/ نعم
- No/ لا
- Don’t know/prefer not to answer/ لا أعرف/ أفضل عدم القول

26. To what extent would you recommend other Syrian refugees to come to Canada?

إلى أي قد مدى تنصح اللاجئين السوريين الآخرين بالقدوم إلى كندا؟

- Not at all ليس البتة /
- A little قليلاً /
- A moderate amount الى حد متوسط /
- Very much كثيراً جداً /
- An extreme amount الى ابعد الحدود /

27. Which neighborhood in the Greater Toronto Area (GTA) do you currently live in?

في أي حي في منطقة تورنتو الكبرى تعيش حالياً؟

- Downtown Toronto/ وسط مدينة تورنتو
- North York/ شمال يورك
- Scarborough and East York / سكاربرو ايست يورك و
- Etobicoke/ ايتوبيكوك
- Mississauga/ ميسيساغا
- Other/ غير ذلك

28. To what extent have your neighbors supported you?

إلى أي مدى حصلت على دعم من جيرانك؟

- Not at all ليس البتة /
- A little قليلاً /
- A moderate amount الى حد متوسط /
- Very much كثيراً جداً /
- An extreme amount الى ابعد الحدود /

29. How many of your friends in this city would you say you feel emotionally close to? Close means with which you feel comfortable talking about personal matters?

كم صديقاً من أصدقائك في هذه المدينة يمكن أن تقول إنك تشعر بأنه مقرب منك؟ وتعني كلمة مقرب هنا شخص تشعر بالارتياح معه في التحدث عن الامور الشخصية؟

- None/ لا يوجد
- One/ واحد
- Two or three/ اثنين أو ثلاثة
- Four or more/ أربعة أو أكثر

30. How satisfied are you with the quality of the friendships you have here?

ما هي نسبة رضاك عن نوعية الصداقات التي لديك هنا؟

- Very satisfied/ راضٍ جداً
- Satisfied/ راضٍ
- Neutral/ Neither satisfied nor unsatisfied/ محايد - لا راضي ولا غير راضي
- Unsatisfied/ غير راضٍ
- Very Unsatisfied/ غير راضي بشدة

31. How often do you need an interpreter for your appointments?

ما هو مدى احتياجك إلى مترجم شفهي لمواعيدك؟

- Always/ دائماً
- Sometimes/ أحياناً
- Never/ أبداً

32. Social Support Cluster:

مجموعة الدعم الاجتماعي:

33. I have close relationships that provide me with a sense of emotional security and well-being.

لدي علاقات قريبة توفر لي الإحساس بالأمان العاطفي والراحة.

- Strongly disagree /	لا أوافق بشدة
- Disagree /	لا أوافق
- Agree /	أوافق
- Strongly agree /	أوافق بشدة

34. There is someone I could talk to about important decisions in my life.

هناك شخص يمكنني التحدث معه عن قرارات مهمة في حياتي.

- Strongly disagree /	لا أوافق بشدة
- Disagree /	لا أوافق
- Agree /	أوافق
- Strongly agree /	أوافق بشدة

35. I have relationships where my competence and skill are recognized.

لدي علاقات حيث يتم الاعتراف بكفاءتي ومهارتي.

- Strongly disagree /	لا أوافق بشدة
- Disagree /	لا أوافق
- Agree /	أوافق
- Strongly agree /	أوافق بشدة

36. I feel part of a group who share my attitudes and beliefs.

أشعر أنني جزء من مجموعة تشارك مواقفي ومعتقداتي.

- Strongly disagree /	لا أوافق بشدة
- Disagree /	لا أوافق
- Agree /	أوافق
- Strongly agree /	أوافق بشدة

37. There are people I can count on in an emergency.

هناك أشخاص يمكنني الاعتماد عليهم في حالات الطوارئ.

- Strongly disagree /	لا أوافق بشدة
- Disagree /	لا أوافق
- Agree /	أوافق
- Strongly agree /	أوافق بشدة

38. Housing Cluster:

The following questions will address your housing

السكن: فيما يلي مجموعة من الأسئلة تتعلق بسكنك.

39. Where do you currently live?

أين تعيش في الوقت الراهن؟

- Shelter/ مأوى
- Subsidized/Social Housing (e.g. Toronto community housing)

الإسكان الاجتماعي (على سبيل المثال إسكان تورنتو المجتمعي)

- Supportive/Transitional/ temporary or substandard housing (e.g. hotel)

سكن مدعوم/ انتقالي/ مؤقت أو أقل من المستوى (أي لا تزال في مركز استقبال – الفندق)

- Living on the streets/ أعيش في الشوارع
- Living in in an institution (e.g. a hospital)

العيش في إحدى المؤسسات (على سبيل المثال مستشفى)

- Rooming house or room in a house/ بيت من غرفة أو غرفة في منزل
- Rented apartment/ شقة بالإيجار
- Rented house/ منزل بالإيجار
- Own my own home/ أمتلك منزلي
- Other
- Rented basement / قبو بالإيجار

40. How many bedrooms are in your home?

كم عدد غرف النوم في منزلك؟

41. What is the number of people living in your home?

كم عدد الأشخاص الذين يعيشون في منزلك؟

42. How much do you pay for rent/mortgage per month?

كم تدفع للإيجار / الرهن العقاري في الشهر؟

- Less than $1000 /	أقل من 1000 دولار
- Between $1000 and $1500 / بين 1000 دولار و 1500 دولار
- Between $1500 and $2000 / بين 1500 دولار و 2000 دولار
- Between $2000 and $2500 / بين 2000 دولار و 2500 دولار
- Between $2500 and $3000 / بين 2500 دولار و3000 دولار
- Between $3000 and $4000 / بين 3000 دولار و 4000 دولار
- More than $4000 / أكثر من 4000 دولار
- Prefer not to answer/ I don’t know / لا أعرف / أفضل عدم الإجابة

43. Overall, how satisfied are you with your housing?

بشكل عام ما هو مدى رضاك عن منزلك؟

- Very satisfied/ راض جداً
- Satisfied/ راضٍ
- Neither satisfied or unsatisfied/ لا راضي ولا غير راضي
- Unsatisfied/ غير راضي
- Very unsatisfied/ غير راضي بشدة

44. Employment and Income Cluster:

The following questions will address your employment and income.

التوظيف والدخل: فيما يلي مجموعة أسئلة تتعلق بعملك ودخلك.

45. Are you currently working?

هل تعمل حاليا؟

- Yes, I am working from home / نعم، أعمل من منزلي
- Yes, I am working in-person / نعم، أعمل خارج المنزل
- No, I am not currently working / لا أعمل حالياً

46. What is your work/occupation in Canada?

ما هو عملك / مهنتك في كندا؟

- Do not work in Canada /	لا أعمل في كندا
- Field of trade and economy/ مجال التجارة والاقتصاد
- Field of industry/ مجال الصناعة
- Field of Agriculture/ مجال الزراعة
- Field of health and medicine/ مجال الصحة والطب
- Field of building and construction/ مجال البناء والاعمار
- Field of education/ مجال التعليم
- Field of engineering/ مجالات هندسية
- Business and finance/ المال والاعمال
- Field of justice/ مجال القضاء
- Field of military/ المجال العسكري
- Craftsmanship/ الاعمال الحرفية
- Field of the arts/ مجال الفنون
- Office employee/ موظف حكومي
- Driver/ سائق
- Other
- Food industry / مجال الطعام/الغذاء

47. What was your work/occupation in Syria?

ما هو عملك/ مهنتك في سوريا؟

- Did not work in Syria/ لم أكن أعمل بسوريا
- Field of trade and economy/ مجال التجارة والاقتصاد
- Field of industry/ مجال الصناعة
- Field of Agriculture/ مجال الزراعة
- Field of health and medicine/ مجال الصحة والطب
- Field of building and construction/ مجال البناء والاعمار
- Field of education/ مجال التعليم
- Field of engineering/ مجالات هندسية
- Business and finance/ المال والاعمال
- Field of justice/ مجال القضاء
- Field of military/ المجال العسكري
- Craftsmanship/ الاعمال الحرفية
- Field of the arts/ مجال الفنون
- Office employee/ موظف حكومي
- Driver/ سائق
- Other
- Food industry / مجال الطعام/الغذاء

48. During the past month, what were your household’s sources of income? Choose all that apply

خلال الشهر الماضي ما هي المصادر لدخل عائلتك؟ رجاء اختيار كل ما ينطبق

- Wages from a job/employment / أجر عن عمل
  - Yes/ نعم
  - No/ لا
- Child Benefits / مزايا الطفل
  - Yes/ نعم
  - No/ لا
- Employment Insurance (EI) / تأمين العمالة
  - Yes/ نعم
  - No/ لا
- Ontario Works or Ontario Disability Support Program (ODSP) /

برنامج أونتاريو لدعم ذوي الإعاقة/ برنامج أونتاريو للمساعدات الاجتماعية

- Yes/ نعم
- No/ لا

- Canadian Emergency Response Benefit (CERB) / إعانة الاستجابة للطوارئ في كندا
  - Yes/ نعم
  - No/ لا
- RAP/ برنامج مساعدة اللاجئين
  - Yes/ نعم
  - No/ لا
- Private sponsor/ كفيل خاص
  - Yes/ نعم
  - No/ لا
- Other/ غير ذلك
  - Yes/ نعم
  - No/ لا

49. During the past year, did you ever eat less because there was not enough food or money for food?

خلال العام الماضي هل اضطررت لتقليل الطعام بسبب عدم وجود طعام كافٍ أو مال لشراء الطعام؟

- Yes/ نعم
- No/	لا

50. During the past year, did you need to visit a food bank because there was not enough food or money for food?

خلال العام الماضي هل اضطررت لزيارة بنك الطعام بسبب عدم وجود طعام كافٍ أو مال لشراء الطعام؟

- Yes/ نعم
- No/ لا

51. Approximately how much money did you have when you first moved to Canada (in CAD)?

تقريبًا كم من المال كان لديك عندما انتقلت إلى كندا لأول مرة (بالدولار الكندي)؟

52. Thinking about your time in Syria before the war, would you say most people would have categorized a household like yours as

عندما كنت تعيش في سوريا قبل الحرب ، هل تعتقد أن معظم الناس قد صنّفوا وضعك العائلي بالتالي:

- Lower income / منخفض الدخل
- Lower middle income / منخفض الى متوسط الدخل
- Middle income / متوسط الدخل
- Upper-middle income / متوسط الى عالي الدخل
- Upper income / عالي الدخل
- Don’t know/prefer not to answer/ لا أعرف أفضل عدم القول

53. In your current condition, here in Canada, would you say most people would categorize a household like yours as

في وضعك الحالي، هنا في كندا، هل تعتقد أن معظم الناس قد يصنّفوا عائلتك بالتالي:

- Lower income / منخفض الدخل
- Lower middle income / منخفض الى متوسط الدخل
- Middle income / متوسط الدخل
- Upper-middle income / متوسط الى عالي الدخل
- Upper income / عالي الدخل
- Don’t know/prefer not to answer/ لا أعرف أفضل عدم القول

54. Education Cluster:

The following set of questions will address your current education.

التعليم: فيما يلي مجموعة من الأسئلة تتعلق بتعليمك الحالي.

55. Please rate your current overall ability in English or French, whichever you are more comfortable with.

يرجى تقييم قدرتك الإجمالية الحالية باللغة الإنجليزية أو الفرنسية، أيهما أكثر راحة لك.

- Excellent/ ممتاز
- Good/ جيد
- Fair/ مقبول
- Poor/ ضعيف
- Very poor/ ضعيف جداً
- Not at all/ لا توجد مطلقاً

56. What is your current Canadian Language Benchmark level?

ما هو مستواك الحالي حسب المقياس الكندي للغة؟

- Level 1 / المستوى ١
- Level 2 / المستوى ٢
- Level 3 / المستوى ٣
- Level 4 / المستوى ٤
- Level 5 / المستوى ٥
- Level 6 / المستوى ٦
- Level 7 / المستوى ٧
- Level 8 / المستوى ٨
- Level 9 / المستوى ٩
- Level 10 / المستوى ١٠
- I don’t know / لا أعرف
- Level 0 / المستوى٠

57. What was your Canadian Language Benchmark level when you first arrived to Canada?

ما كان مستواك حسب المقياس الكندي للغة الخاص بك عندما وصلت لأول مرة إلى كندا؟

- Level 1 / المستوى ١
- Level 2 / المستوى ٢
- Level 3 / المستوى ٣
- Level 4 / المستوى ٤
- Level 5 / المستوى ٥
- Level 6 / المستوى ٦
- Level 7 / المستوى ٧
- Level 8 / المستوى ٨
- Level 9 / المستوى ٩
- Level 10 / المستوى ١٠
- I don’t know / لا أعرف
- Level 0 / المستوى٠

Language Classes Cluster:

58. Have you taken at least one government-funded English/French language class (LINC)?

هل أخذت صف واحد على الأقل في دورات اللغة الإنجليزية أو الفرنسية الممولة من الحكومة؟

- Yes, i am currently taking language classes online / نعم، حالياً عبر الانترنت
- Yes, i have previously taken language classes / نعم أخذت دورات اللغة مسبقاً
- No, i did not take language classes / لا لم آخد دورات اللغة

(ONLY ASK THE REST OF THIS CLUSTER IF THEY HAVE TAKEN A LANGUAGE CLASS)

59. Approximately how many months of English/French language classes have you taken in total?

ما هو إجمالي عدد الأشهر التي أمضيتها في دورات اللغة الانجليزية او الفرنسية؟

60. During these months, on average, how many hours of language classes do you attend per week?

خلال تلك الأشهر كم متوسط عدد الساعات التي تحضرها في الأسبوع في دورات اللغة ؟ ـــــــــــــــــــــــــــ

61. Please rate your overall satisfaction with these language classes.

يرجى تقييم مدى رضاك العام عن دروس اللغة.

- Very satisfied/ راض جداً
- Satisfied/ راضٍ
- Neither satisfied or unsatisfied/ لا راضي ولا غير راضي
- Unsatisfied/ غير راضي
- Very unsatisfied/ غير راضي بشدة

62. What changes would you like to see within the language classes? Please select all that apply:

ما هي التغييرات التي تود أن تراها في صفوف اللغة؟ يرجى اختيار كل ما ينطبق:

- More students that speak my native language

عدد طلاب أكثر الذين يتحدثون لغتي الأم

- Yes/ نعم
- No/ لا
- Less students that speak my native language

عدد طلاب أقل الذين يتحدثون لغتي الأم

- Yes/ نعم
- No/ لا
- Smaller class sizes

أحجام صفوف أصغر

- Yes/ نعم
- No/ لا
- Larger class sizes

أحجام صفوف أكبر

- Yes/ نعم
- No/ لا
- Change schedule/offer classes at different times

تغيير الجدول / عرض محاضرات في أوقات مختلفة

- Yes/ نعم
- No/ لا
- Different curriculum/teaching styles

مناهج وأساليب تدريس مختلفة

- Yes/ نعم
- No/ لا
- More discussion within classes/more oppurtunities to practice

**مناقشات أكثر داخل الصفوف / فرص أكثر للتدريب والممارسة**

- Yes/ نعم
- No/ لا
- Other /	غير
- Online class accessibility/eligibility/ إمكانية الوصول أو المشاركة بالصفوف عبر الانترنت
  - Yes/ نعم
  - No/ لا

63. Did you experience barriers in accessing language classes, such as too far, too expensive, can’t find child care?

هل صادفتك عقبات للحصول على دروس تعلّم اللغة، مثل مكلفة للغاية، بعيدة جداً، الحصول على رعاية للأطفال؟

- Yes/ نعم
- No/ لا

64. What barriers, if any, have you experienced in accessing the language classes? Choose all that apply.

ما هي العقبات، إن وجدت، التي تعرضت لها للحصول على دروس تعلّم اللغة ؟ (رجاء وضع علامة أمام كل ما ينطبق عليه)

- Too expensive/ مكلفة للغاية
  - Yes/ نعم
  - No/ لا
- Can't find appropriate or affordable child care for my children

لا أستطيع الحصول على رعاية مناسبة أو رخيصة للأطفال

- Yes/ نعم
- No/ لا
- Not enough time because of other obligations/ لا يوجد وقت كافٍ بسبب التزامات أخرى
  - Yes/ نعم
  - No/ لا
- Too far/ بعيدة جداً
  - Yes/ نعم
  - No/ لا
- Other, please specify/ غير ذلك

65. Subjective Cluster:

Please complete the following statements with the option that best represents your experience.

يرجى اكمال العبارات التالية أدناه مع الخيار الذي يمثل تجربتك على أفضل وجه.

66. The cost of living in Canada is:

تكلفة المعيشة في كندا هي:

- Much more expensive than I expected pre-migration / أغلى بكثير مما توقعت قبل اللجوء
- Slightly more expensive than I expected pre-migration / أغلى بقليل مما توقعت قبل اللجوء
- As expensive as I expected pre-migration / غالي كما توقعت قبل اللجوء
- Slightly less expensive than I expected pre-migration / أقل تكلفة بقليل مما كنت أتوقع قبل اللجوء
- Much less expensive than I expected pre-migration / أقل تكلفة بكثير مما توقعت قبل اللجوء

67. The weather in Canada is:

الطقس في كندا هو:

- Much colder than I expected pre-migration /أبرد بكثير مما توقعت قبل اللجوء
- Slightly colder than I expected pre-migration / أبرد بقليل مما توقعته قبل اللجوء
- As cold as I expected pre-migration / بارد كما توقعت قبل اللجوء
- Slightly warmer than I expected pre-migration / أكثر دفئًا بقليل مما كنت أتوقع قبل اللجوء
- Much warmer than I expected pre-migration / أكثر دفئًا بكثير مما توقعت قبل اللجوء

68. The people of Canada are:

إن شعب كندا هم:

- Much friendlier than I expected pre-migration / أكثر ودية بكثير مما كنت أتوقع قبل اللجوء
- Slightly friendlier than I expected pre-migration / أكثر ودية بقليل مما كنت أتوقع قبل اللجوء
- As friendly as I expected pre-migration / وديون كما توقعت قبل اللجوء
- Slightly less friendly than I expected pre-migration / أقل ودية مما كنت أتوقع قبل اللجوء
- Much less friendly than I expected pre-migration / أقل ودية بكثير مما توقعت قبل اللجوء

69. How difficult has the transition into Canada been for you?

ما مدى صعوبة الانتقال إلى كندا و التأقلم بالنسبة لك؟

- Very difficult / صعب جدا
- Difficult / صعب
- Neutral / محايد/عادي
- Not difficult / غير صعب
- Not difficult at all / غير صعب على الإطلاق

70. How difficult do you think the transition into Canada has been for your children?

ما مدى صعوبة الانتقال إلى كندا و التأقلم بالنسبة لأطفالك؟

- Very difficult / صعب جدا
- Difficult / صعب
- Neutral / محايد/عادي
- Not difficult / غير صعب
- Not difficult at all / غير صعب على الإطلاق

71. To what extent do you feel that your living in Canada is a sacrifice you make for your children everyday?

إلى أي مدى تشعر أن حياتك في كندا هي تضحية تقدمها لأطفالك كل يوم؟

- Not at all ليس البتة /
- A little قليلاً /
- A moderate amount الى حد متوسط /
- Very much كثيراً جداً /
- An extreme amount الى ابعد الحدود /

72. Do you find Canadians to be welcoming and accepting?

هل تجد الكنديين مرحبين ومتقبلين؟

- Almost always/ تقريباً دائماً
- Usually/ عادة
- Only sometimes/ أحياناً فقط
- Never/ أبداً

73. Do you feel that Canadians treat you with respect?

هل تشعر أن الكنديين يعاملونك باحترام؟

- Almost always/ تقريباً دائماً
- Usually/ عادة
- Only sometimes/ أحياناً فقط
- Never/ أبداً

74. How would you describe your sense of belonging to:

كيف تصف شعورك بالانتماء إلى:

Your neighborhood

حيِّك

- Very Strong/ قوي جداً
- Strong/ قوي
- Weak/ ضعيف
- Very weak/ ضعيف جداً

75. Canada

كندا

- Very Strong/ قوي جداً
- Strong/ قوي
- Weak/ ضعيف
- Very weak/ ضعيف جداً

76. Your ethnic community or cultural group here in Canada

مجتمعك الاثني أو العرقي أو مجموعتك الثقافية هنا في كندا

- Very Strong/ قوي جداً
- Strong/ قوي
- Weak/ ضعيف
- Very weak/ ضعيف جداً

77. How safe from physical abuse do you think women are in Canada?

إلى أي مدى تعتقد أن المرأة آمنة من الاعتداء الجسدي في كندا؟

- Not at all ليس البتة /
- A little قليلاً /
- A moderate amount الى حد متوسط /
- Very much كثيراً جداً /
- An extreme amount الى ابعد الحدود /

78. How safe from physical abuse do you think women are in the Middle East?

إلى أي مدى تعتقد أن المرأة آمنة من الاعتداء الجسدي في الشرق الأوسط؟

- Not at all ليس البتة /
- A little قليلاً /
- A moderate amount الى حد متوسط /
- Very much كثيراً جداً /
- An extreme amount الى ابعد الحدود /

79. How safe from verbal and emotional abuse do you think women are in Canada?

إلى أي مدى تعتقد أن المرأة آمنة من الاعتداء اللفظي و المعنوي في كندا؟

- Not at all ليس البتة /
- A little قليلاً /
- A moderate amount الى حد متوسط /
- Very much كثيراً جداً /
- An extreme amount الى ابعد الحدود /

80. How safe from verbal and emotional abuse do you think women are in the Middle East?

إلى أي مدى تعتقد أن المرأة آمنة من الاعتداء اللفظي و المعنوي في الشرق الأوسط؟

- Not at all ليس البتة /
- A little قليلاً /
- A moderate amount الى حد متوسط /
- Very much كثيراً جداً /
- An extreme amount الى ابعد الحدود /

Perceived Security Cluster:

81. Do you feel safe in your home with the people you live with?

هل تشعر بالأمان في منزلك مع الأشخاص الذين تعيش معهم؟

- Yes/ نعم
- Somewhat/ إلى حد ما
- No/ لا
- Not sure/ prefer not to answer/ غير متأكد\أفضل عدم الإجابة

82. Do you feel safe in your community?

هل تشعر بالأمان في مجتمعك؟

- Yes/ نعم
- Somewhat/ إلى حد ما
- No/ لا
- Not sure/ prefer not to answer/ غير متأكد\أفضل عدم الإجابة

Attitudes towards Schools Cluster:

المواقف تجاه المدارس:

83. Do you have one or more children who attend/attended school in Canada?

هل لديك طفل أو أطفال يذهبون أو ذهبوا إلى المدرسة في كندا؟

- Yes / نعم
- No / لا

(ONLY ASK THE REST OF THE CLUSTER IF THEY REPLY YES)

84. Please rate your overall satisfaction with your child(ren)’s school(s) in Canada.

يرجى تقييم مدى رضاك بشكل عام عن مدرسة أطفالك في كندا.

- Very satisfied/ راض جداً
- Satisfied/ راضٍ
- Neither satisfied or unsatisfied/ لا راضي ولا غير راضي
- Unsatisfied/ غير راضي
- Very unsatisfied/ غير راضي بشدة

85. How often do you as a parent face discrimination in your child(ren)’s school events?

ما هو مدى مواجهتك التمييز كوالد/والدة في الأحداث المدرسية لأطفالك؟

- Never ابداً /
- Seldom نادراً /
- Quite often احياناً /
- Very often في معظم الأحيان /
- Always دائماً /

86. How involved are you with your child(ren)’s education (e.g. solving homework, keeping track of examination dates and grades, etc.)?

ما مدى ارتباطك بتعليم أطفالك (حل الواجبات المنزلية، اتباع مواعيد ودرجات الامتحانات، الخ)؟

- Not at all ليس البتة /
- A little قليلاً /
- A moderate amount الى حد متوسط /
- Very much كثيراً جداً /
- An extreme amount الى ابعد الحدود /

87. Family Functioning Cluster:

The following questions will address your family relationships.

مجموعة أداء العائلة:

ستتناول الأسئلة التالية العلاقات الأسرية.

88. Planning family activities is difficult because we misunderstand each other.

إن التخطيط للأنشطة العائلية أمر صعب لأننا نسيء فهم بعضنا البعض.

- Strongly agree / أوافق بشدة
- Agree / أوافق
- Disagree / لا أوافق
- Strongly disagree / لا أوافق بشدة

89. In time of crisis we can turn to each other for support.

عند الأزمات ، يمكننا اللجوء إلى بعضنا البعض للحصول على الدعم.

- Strongly agree / أوافق بشدة
- Agree / أوافق
- Disagree / لا أوافق
- Strongly disagree / لا أوافق بشدة

90. We cannot talk to each other about sadness we feel.

لا يمكننا التحدث مع بعضنا البعض عن الحزن الذي نشعر به.

- Strongly agree / أوافق بشدة
- Agree / أوافق
- Disagree / لا أوافق
- Strongly disagree / لا أوافق بشدة

91. Individuals are accepted for what they are.

يتم قبول الأفراد على ما هم عليه.

- Strongly agree / أوافق بشدة
- Agree / أوافق
- Disagree / لا أوافق
- Strongly disagree / لا أوافق بشدة

92. We avoid discussing our fears and concerns.

نتجنب مناقشة مخاوفنا واهتماماتنا.

- Strongly agree / أوافق بشدة
- Agree / أوافق
- Disagree / لا أوافق
- Strongly disagree / لا أوافق بشدة

93. We can express feelings to each other.

يمكننا التعبير عن المشاعر لبعضنا البعض.

- Strongly agree / أوافق بشدة
- Agree / أوافق
- Disagree / لا أوافق
- Strongly disagree / لا أوافق بشدة

94. There are lots of bad feelings in the family.

هناك الكثير من المشاعر السيئة في الأسرة.

- Strongly agree / أوافق بشدة
- Agree / أوافق
- Disagree / لا أوافق
- Strongly disagree / لا أوافق بشدة

95. We feel accepted for what we are.

نشعر بالقبول على ما نحن عليه.

- Strongly agree / أوافق بشدة
- Agree / أوافق
- Disagree / لا أوافق
- Strongly disagree / لا أوافق بشدة

96. Making decisions is a problem for our family.

يشكّل اتخاذ القرارات مشكلة لعائلتنا.

- Strongly agree / أوافق بشدة
- Agree / أوافق
- Disagree / لا أوافق
- Strongly disagree / لا أوافق بشدة

97. We are able to make decisions about how to solve problems.

نحن قادرون على اتخاذ القرارات حول كيفية حل المشاكل.

- Strongly agree / أوافق بشدة
- Agree / أوافق
- Disagree / لا أوافق
- Strongly disagree / لا أوافق بشدة

98. We don't get along well together.

نحن لا نتفق معًا بشكل جيد.

- Strongly agree / أوافق بشدة
- Agree / أوافق
- Disagree / لا أوافق
- Strongly disagree / لا أوافق بشدة

99. We confide in each other.

نحن نثق في بعضنا البعض.

- Strongly agree / أوافق بشدة
- Agree / أوافق
- Disagree / لا أوافق
- Strongly disagree / لا أوافق بشدة

# 100. Family Roles Cluster:

The following questions will address the roles within your family members.

أدوار الأسرة: تتناول الأسئلة التالية أدوار أفراد عائلتك.

101. Where did you get married?

أين تزوجت؟

- Syria / سوريا
- Host country / بلد مضيف (مثل لبنان، تركيا)
- Canada / كندا

102. Did the roles of your family members change from when you were living in Syria? Such as controlling the finances and the main caregiver for the children

هل تغيرت أدوار أفراد أسرتك عما كانت عليه عندما كنت تعيش في سوريا؟ مثل من يتحكم في الشؤون المالية للعائلة و مقدم الرعاية الرئيسي للأطفال

- Yes / نعم
- No / لا
- I don’t know / لا أعرف

103. When you were living in Syria/host country, who was the main caregiver to the children?

عندما كنت تعيش في سوريا / البلد المضيف ، من كان مقدم الرعاية الرئيسي للأطفال؟

- Mother / الأم
- Father / الآب
- Both mother and father / الأم و الأب
- Other, please specify / شخص آخر

104. Currently, who is the main caregiver to the children?

حاليا، من هو مقدم الرعاية الرئيسي للأطفال؟

- Mother / الأم
- Father / الآب
- Both mother and father / الأم و الأب
- Other, please specify / شخص آخر

105. When you were living in Syria/host country, who made the day-to-day decisions for the family?

عندما كنت تعيش في سوريا/ البلد المضيف ، من الذي كان يتخذ القرارات اليومية للعائلة؟

- Mother / الأم
- Father / الآب
- Both mother and father / الأم و الأب
- Other, please specify / شخص آخر

106. Currently, who makes the day-to-day decisions for the family?

حاليا، من الذي يتخذ القرارات اليومية للعائلة؟

- Mother / الأم
- Father / الآب
- Both mother and father / الأم و الأب
- Other, please specify / شخص آخر

107. When you were living in Syria/host country, who controlled the finances for the family?

عندما كنت تعيش في سوريا / البلد المضيف ، من الذي كان يتحكم في الشؤون المالية للعائلة ؟

- Mother / الأم
- Father / الآب
- Both mother and father / الأم و الأب
- Other, please specify / شخص آخر

108. Currently, who controls the finances for the family?

حاليا، من الذي يتحكم في الشؤون المالية للعائلة؟

- Mother / الأم
- Father / الآب
- Both mother and father / الأم و الأب
- Other, please specify / شخص آخر

109. When you were living in Syria/host country, who made the major life decisions for the family?

عندما كنت تعيش في سوريا / البلد المضيف ، من الذي كان يتخذ قرارات الحياة الرئيسية للعائلة؟

- Mother / الأم
- Father / الآب
- Both mother and father / الأم و الأب
- Other, please specify / شخص آخر

110. Currently, who makes the major life decisions for the family?

حاليا، من الذي يتخذ قرارات الحياة الرئيسية للعائلة؟

- Mother / الأم
- Father / الآب
- Both mother and father / الأم و الأب
- Other, please specify / شخص آخر

111. General Health Cluster:

The following questions will address your health status

الصحة: الاسئلة الآتية سوف تتعلق بحالتك الصحية

112. Do you have a family doctor?

هل لديك طبيب عائلة؟

- Yes/ نعم
- No/ لا
- Don't know/ لا أعرف

113. Do you smoke cigarettes?

هل تدخن السجائر؟

- Yes/ نعم
- No/ لا

114. How many cigarettes per day? _____

كم سيجارة في اليوم؟ ـــــــــــــــــ

115. Do you smoke argile/shisha?

هل تدخن أرغليه/شيشا؟

- Yes/ نعم
- No/ لا

116. How often do you smoke argile/shisha?

كم مرة تدخن أرغليه/شيشا ؟

- Several times a day / عدة مرات في اليوم
- Once everyday / مرة في اليوم
- Few times per week / عدة مرات في الاسبوع
- Once per week / مرة في الاسبوع
- Few times per month / عدة مرات في الشهر
- Other/ غير ذلك

117. Do you drink alcohol?

هل تشرب الخمر/الكحول؟

- Yes/ نعم
- No/ لا

118. How often do you drink alcohol

كم مرة تشرب الخمر/الكحول؟

- Several times a day / عدة مرات في اليوم
- Once everyday / مرة في اليوم
- Few times per week / عدة مرات في الاسبوع
- Once per week / مرة في الاسبوع
- Few times per month / عدة مرات في الشهر
- Other/ غير ذلك

119. In general, would you say your physical health is:

بصورة عامة، كيف ترى صحتك الجسدية؟

- Excellent/ ممتازة
- Very good/ جيدة جداً
- Good/ جيدة
- Fair/ لا بأس بها
- Poor/ سيئة

120. Have you ever tested positive for COVID-19?

هل حصلت على نتيجة إيجابية لاختبار فيروس كورونا-١٩ مسبقاً ؟

- Yes / نعم
- No / لا
- I don’t know/ prefer not to answer / لا أعرف / أفضل عدم الإجابة

121. If any, how severe were your COVID-19 symptoms, such as coughing, shortness of breath, fever, etc?

إذا شعرت بأعراض فيروس كورونا-١٩ كالسعال أو الحمى أو ضیق التنفس الخ، ما كانت مدى خطورة أو شدة تلك الأعراض؟

- Not at all ليس البتة /
- A little قليلاً /
- A moderate amount الى حد متوسط /
- Very much كثيراً جداً /
- An extreme amount الى ابعد الحدود /
- I did not experience any symptoms / لم أشعر بأي أعراض

122. Did any of your close friends or family members get seriously ill or pass away because of COVID-19?

هل توفي أحد من أصدقائك المقربين أو أفراد عائلتك أو أصيب بمرض خطير بسبب الإصابة بفيروس كورونا-١٩ ؟

- Yes / نعم
- No / لا
- I don’t know/ prefer not to answer / لا أعرف / أفضل عدم الإجابة

123. Are you going to take the vaccine for the Coronavirus-19 when it becomes available to you?

هل ستأخذ لقاح لفيروس كورونا-١٩ عندما يصبح متوفر؟

- Yes / نعم
- No / لا
- I don’t know / لا أعرف
- Prefer not to answer/ أفضل عدم الإجابة
- I have already taken the Coronavirus-19 vaccine / لقد أخذت اللقاح مسبقاً

Fear of Coronavirus-19 Cluster:

الخوف من فيروس الكورونا- ١٩:

124. I am most afraid of coronavirus-19.

أنا خائف من فيروس كورونا-١٩ لأقصى حدّ

- Strongly disagree / لا أوافق بشدة
- Disagree / لا أوافق
- Neither agree nor disagree / محايد
- Agree / أوافق
- Strongly agree / أوافق بشدة

125. It makes me uncomfortable to think about coronavirus-19.

التفكير بفيروس كورونا-١٩ يشعرني بعدم الارتياح

- Strongly disagree / لا أوافق بشدة
- Disagree / لا أوافق
- Neither agree nor disagree / محايد
- Agree / أوافق
- Strongly agree / أوافق بشدة

126. My hands become clammy when I think about coronavirus-19.

أشعر بتعرق في كفيّ عندما أفكر بفيروس كورونا-١٩

- Strongly disagree / لا أوافق بشدة
- Disagree / لا أوافق
- Neither agree nor disagree / محايد
- Agree / أوافق
- Strongly agree / أوافق بشدة

127. I am afraid of losing my life because of coronavirus-19.

أخشى أن أفقد حياتي بسبب فيروس كورونا-١٩

- Strongly disagree / لا أوافق بشدة
- Disagree / لا أوافق
- Neither agree nor disagree / محايد
- Agree / أوافق
- Strongly agree / أوافق بشدة

128. When watching news and stories about coronavirus-19 on social media, I become nervous or anxious.

أ ينتابني القلق أو التوتر عندما أشاهد أخبارًا أو قصصًا عن فيروس كورونا-١٩

- Strongly disagree / لا أوافق بشدة
- Disagree / لا أوافق
- Neither agree nor disagree / محايد
- Agree / أوافق
- Strongly agree / أوافق بشدة

129. I cannot sleep because I’m worrying about getting coronavirus-19.

لا يمكنني النوم بسبب قلقي من الإصابة بعدوى فيروس كورونا-١٩

- Strongly disagree / لا أوافق بشدة
- Disagree / لا أوافق
- Neither agree nor disagree / محايد
- Agree / أوافق
- Strongly agree / أوافق بشدة

130. My heart races or palpitates when I think about getting coronavirus-19.

تتسارع دقات قلبي عندما أفكر بالإصابة بعدوى فيروس كورونا-١٩

- Strongly disagree / لا أوافق بشدة
- Disagree / لا أوافق
- Neither agree nor disagree / محايد
- Agree / أوافق
- Strongly agree / أوافق بشدة

131. Mental Health Cluster:

The following questions will address your mental health status

الصحة النفسية: فيما يلي مجموعة من الأسئلة تتعلق بحالة صحتك النفسية

132. In general would you say your mental health is?

بشكل عام هل تقول إن صحتك النفسية:

- Excellent/ ممتازة
- Very good/ جيدة جداً
- Good/ جيدة
- Fair/ مقبولة
- Poor/ سيئة

133. If you struggle(d) with mental health issues, who do/did you contact for this/these problem(s)/concern(s)? (Please select all that apply):

إذا كنت تعاني من مشاكل نفسية، من من هذا القائمة ممكن لك ان تعبر له عن مشاكلك او مخاوفك؟ يرجى اختيار كل ما ينطبق:

- Psychiatrist/ Psychologist

طبيب نفسي/ عالم نفسي

- Yes/ نعم
- No/ لا
- Social worker / Counselor

عامل اجتماعي /مستشار

- Yes/ نعم
- No/ لا
- Minister/imam/clergy / Spiritual healer / Traditional healer/medium

رجل دين/ امام/ معالج الروحي / معالج تقليدي

- Yes/ نعم
- No/ لا
- Friends / family members

اصحاب/ افراد العائلة

- Yes/ نعم
- No/ لا
- Medical doctor

طبيب

- Yes/ نعم
- No/ لا
- Private sponsor

كفيل الخاص

- Yes/ نعم
- No/ لا
- No one

لا أحد

- Yes/ نعم
- No/ لا
- Other/ غير ذلك

134. How would you rate your quality of life?

كيف تقيم جودة حياتك؟

- Very poor	ضعيف جداً /
- Poor	ضعيف/
- Neither poor nor good متوسط /
- Good	جيد /
- Very good جيد جداً /

# 135. PTSD Cluster:

In your life, have you ever had any experience that was so frightening, horrible, or upsetting that, in the past month, you:

هل تعرضت خلال حياتك لأي تجربة كانت مرعبة أو مخيفة أو محزنة لدرجة أنك في الشهر الماضي:

136. Have had nightmares about it or thought about it when you did not want to?

تعرضت لكوابيس بخصوصها أو فكرت فيها على غير رغبتك؟

- Yes/ نعم
- No/ لا

137. Tried hard not to think about it or went out of your way to avoid situations that reminded you of it?

حاولت جاهدا عدم التفكير فيها وفعلت كل ما بوسعك لتجنب المواقف التي تذكرك بها؟

- Yes/ نعم
- No/ لا

138. Were constantly on guard, watchful, or easily startled?

كنت دائماً حذراً أو منتبهاً أو سهل الانزعاج؟

- Yes/ نعم
- No/ لا

139. Felt numb or detached from others, activities, or your surroundings?

شعرت بفقدان الحس أو الانفصال عن الآخرين أو الأنشطة أو البيئة المحيطة؟

- Yes/ نعم
- No/ لا

140. DASS 21 Cluster:

Please read each statement and choose an option which indicates how much the statement applied to you over the past week.

اقرأ كل من النصوص التالية ثم اختر الرقم ٠،١،٢،٣ الذي يبين درجة انطباق هذا الشعور عليك في الأسبوع الماضي.

141. I found it hard to wind down

وجدت صعوبة في الاسترخاء و الراحة

- Did not apply to me at all / لا ينطبق علي بتاتاً
- Applied to me to some degree, or some of the time / ينطبق علي بعض الشي أو قليلاً من الأوقات
- Applied to me to a considerable degree, or a good part of the time / ينطبق علي بدرجة ملحوظة أو بعض الأوقات
- Applied to me very much, or most of the time / ينطبق علي كثيراً جداً أو معظم الأوقات

142. I was aware of dryness of my mouth

شعرت بجفاف في حلقي

- Did not apply to me at all / لا ينطبق علي بتاتاً
- Applied to me to some degree, or some of the time / ينطبق علي بعض الشي أو قليلاً من الأوقات
- Applied to me to a considerable degree, or a good part of the time / ينطبق علي بدرجة ملحوظة أو بعض الأوقات
- Applied to me very much, or most of the time / ينطبق علي كثيراً جداً أو معظم الأوقات

143. I couldn’t seem to experience any positive feeling at all

لم يبدو ان بإمكاني الإحساس بمشاعر إيجابية على الإطلاق

- Did not apply to me at all / لا ينطبق علي بتاتاً
- Applied to me to some degree, or some of the time / ينطبق علي بعض الشي أو قليلاً من الأوقات
- Applied to me to a considerable degree, or a good part of the time / ينطبق علي بدرجة ملحوظة أو بعض الأوقات
- Applied to me very much, or most of the time / ينطبق علي كثيراً جداً أو معظم الأوقات

144. I experiences breathing difficulty (eg, excessively rapid breathing, breathlessness in the absence of physical exertion)

شعرت بصعوبة في التنفس (شدة التنفس السريع، اللهثان بدون القيام بمجهود جسدي مثلاً)

- Did not apply to me at all / لا ينطبق علي بتاتاً
- Applied to me to some degree, or some of the time / ينطبق علي بعض الشي أو قليلاً من الأوقات
- Applied to me to a considerable degree, or a good part of the time / ينطبق علي بدرجة ملحوظة أو بعض الأوقات
- Applied to me very much, or most of the time / ينطبق علي كثيراً جداً أو معظم الأوقات

145. I found it difficult to work up the initiative to do things

وجدت صعوبة في أخذ المبادرة بعمل الأشياء

- Did not apply to me at all / لا ينطبق علي بتاتاً
- Applied to me to some degree, or some of the time / ينطبق علي بعض الشي أو قليلاً من الأوقات
- Applied to me to a considerable degree, or a good part of the time / ينطبق علي بدرجة ملحوظة أو بعض الأوقات
- Applied to me very much, or most of the time / ينطبق علي كثيراً جداً أو معظم الأوقات

146. I tended to over-react to situations

كنت أميل إلى ردة فعل مفرطة للظروف والأحداث

- Did not apply to me at all / لا ينطبق علي بتاتاً
- Applied to me to some degree, or some of the time / ينطبق علي بعض الشي أو قليلاً من الأوقات
- Applied to me to a considerable degree, or a good part of the time / ينطبق علي بدرجة ملحوظة أو بعض الأوقات
- Applied to me very much, or most of the time / ينطبق علي كثيراً جداً أو معظم الأوقات

147. I experienced trembling (eg, in the hands)

شعرت برجفة (باليدين مثلاً)

- Did not apply to me at all / لا ينطبق علي بتاتاً
- Applied to me to some degree, or some of the time / ينطبق علي بعض الشي أو قليلاً من الأوقات
- Applied to me to a considerable degree, or a good part of the time / ينطبق علي بدرجة ملحوظة أو بعض الأوقات
- Applied to me very much, or most of the time / ينطبق علي كثيراً جداً أو معظم الأوقات

148. I felt that I was using a lot of nervous energy

شعرت بأنني أستهلك الكثير في الطاقة العصبية (شعرت بأنني أستهلك الكثير من قدرتي على تحمل التوتر العصبي)

- Did not apply to me at all / لا ينطبق علي بتاتاً
- Applied to me to some degree, or some of the time / ينطبق علي بعض الشي أو قليلاً من الأوقات
- Applied to me to a considerable degree, or a good part of the time / ينطبق علي بدرجة ملحوظة أو بعض الأوقات
- Applied to me very much, or most of the time / ينطبق علي كثيراً جداً أو معظم الأوقات

149. I was worried about situations in which I might panic and make a fool of myself

كنت خائفاً من مواقف قد أفقد فيها السيطرة على أعصابي واسبب إحراجاً لنفسي

- Did not apply to me at all / لا ينطبق علي بتاتاً
- Applied to me to some degree, or some of the time / ينطبق علي بعض الشي أو قليلاً من الأوقات
- Applied to me to a considerable degree, or a good part of the time / ينطبق علي بدرجة ملحوظة أو بعض الأوقات
- Applied to me very much, or most of the time / ينطبق علي كثيراً جداً أو معظم الأوقات

150. I felt that I had nothing to look forward to

شعرت بأن ليس لدي أي شيء أتطلع إليه

- Did not apply to me at all / لا ينطبق علي بتاتاً
- Applied to me to some degree, or some of the time / ينطبق علي بعض الشي أو قليلاً من الأوقات
- Applied to me to a considerable degree, or a good part of the time / ينطبق علي بدرجة ملحوظة أو بعض الأوقات
- Applied to me very much, or most of the time / ينطبق علي كثيراً جداً أو معظم الأوقات

151. I found myself getting agitated

شعرت بأنني مضطرب ومنزعج

- Did not apply to me at all / لا ينطبق علي بتاتاً
- Applied to me to some degree, or some of the time / ينطبق علي بعض الشي أو قليلاً من الأوقات
- Applied to me to a considerable degree, or a good part of the time / ينطبق علي بدرجة ملحوظة أو بعض الأوقات
- Applied to me very much, or most of the time / ينطبق علي كثيراً جداً أو معظم الأوقات

152. I found it difficult to relax

أجد صعوبة في الاسترخاء

- Did not apply to me at all / لا ينطبق علي بتاتاً
- Applied to me to some degree, or some of the time / ينطبق علي بعض الشي أو قليلاً من الأوقات
- Applied to me to a considerable degree, or a good part of the time / ينطبق علي بدرجة ملحوظة أو بعض الأوقات
- Applied to me very much, or most of the time / ينطبق علي كثيراً جداً أو معظم الأوقات

153. I felt down-hearted and blue

شعرت بالحزن والغم

- Did not apply to me at all / لا ينطبق علي بتاتاً
- Applied to me to some degree, or some of the time / ينطبق علي بعض الشي أو قليلاً من الأوقات
- Applied to me to a considerable degree, or a good part of the time / ينطبق علي بدرجة ملحوظة أو بعض الأوقات
- Applied to me very much, or most of the time / ينطبق علي كثيراً جداً أو معظم الأوقات

154. I was intolerant of anything that kept me from getting on with what I was doing

كنت لا أستطع تحمل أي شيء يحول بيني وبين ما أرغب في القيام به

- Did not apply to me at all / لا ينطبق علي بتاتاً
- Applied to me to some degree, or some of the time / ينطبق علي بعض الشي أو قليلاً من الأوقات
- Applied to me to a considerable degree, or a good part of the time / ينطبق علي بدرجة ملحوظة أو بعض الأوقات
- Applied to me very much, or most of the time / ينطبق علي كثيراً جداً أو معظم الأوقات

155. I felt I was close to panic

شعرت بأنني على وشك الوقوع في حالة من الرعب المفاجئ بدون سبب

- Did not apply to me at all / لا ينطبق علي بتاتاً
- Applied to me to some degree, or some of the time / ينطبق علي بعض الشي أو قليلاً من الأوقات
- Applied to me to a considerable degree, or a good part of the time / ينطبق علي بدرجة ملحوظة أو بعض الأوقات
- Applied to me very much, or most of the time / ينطبق علي كثيراً جداً أو معظم الأوقات

156. I was unable to become enthusiastic about anything

فقدت الشعور بالحماس لأي شيء

- Did not apply to me at all / لا ينطبق علي بتاتاً
- Applied to me to some degree, or some of the time / ينطبق علي بعض الشي أو قليلاً من الأوقات
- Applied to me to a considerable degree, or a good part of the time / ينطبق علي بدرجة ملحوظة أو بعض الأوقات
- Applied to me very much, or most of the time / ينطبق علي كثيراً جداً أو معظم الأوقات

157. I felt I wasn’t worth much as a person

شعرت بأن قيمتي قليلة كشخص

- Did not apply to me at all / لا ينطبق علي بتاتاً
- Applied to me to some degree, or some of the time / ينطبق علي بعض الشي أو قليلاً من الأوقات
- Applied to me to a considerable degree, or a good part of the time / ينطبق علي بدرجة ملحوظة أو بعض الأوقات
- Applied to me very much, or most of the time / ينطبق علي كثيراً جداً أو معظم الأوقات

158. I felt that I was rather touchy

شعرت بأنني أميل إلى الغيظ بسرعة

- Did not apply to me at all / لا ينطبق علي بتاتاً
- Applied to me to some degree, or some of the time / ينطبق علي بعض الشي أو قليلاً من الأوقات
- Applied to me to a considerable degree, or a good part of the time / ينطبق علي بدرجة ملحوظة أو بعض الأوقات
- Applied to me very much, or most of the time / ينطبق علي كثيراً جداً أو معظم الأوقات

159. I was aware of the action of my heart in the absence of physical exertion (eg, sense of heart rate increase, heart missing a beat)

شعرت بضربات قلبي بدون مجهود جسدي (زيادة في معدل الدقات، أو غياب دقة قلب مثلاً)

- Did not apply to me at all / لا ينطبق علي بتاتاً
- Applied to me to some degree, or some of the time / ينطبق علي بعض الشي أو قليلاً من الأوقات
- Applied to me to a considerable degree, or a good part of the time / ينطبق علي بدرجة ملحوظة أو بعض الأوقات
- Applied to me very much, or most of the time / ينطبق علي كثيراً جداً أو معظم الأوقات

160. I felt scared without any good reason

شعرت بالخوف بدون أي سبب وجيه

- Did not apply to me at all / لا ينطبق علي بتاتاً
- Applied to me to some degree, or some of the time / ينطبق علي بعض الشي أو قليلاً من الأوقات
- Applied to me to a considerable degree, or a good part of the time / ينطبق علي بدرجة ملحوظة أو بعض الأوقات
- Applied to me very much, or most of the time / ينطبق علي كثيراً جداً أو معظم الأوقات

161. I felt that life was meaningless

شعرت بأن الحياة ليس لها معنى

- Did not apply to me at all / لا ينطبق علي بتاتاً
- Applied to me to some degree, or some of the time / ينطبق علي بعض الشي أو قليلاً من الأوقات
- Applied to me to a considerable degree, or a good part of the time / ينطبق علي بدرجة ملحوظة أو بعض الأوقات
- Applied to me very much, or most of the time / ينطبق علي كثيراً جداً أو معظم الأوقات

162. Healthcare Services Cluster:

مجموعة خدمات الرعاية الصحية:

163. Do you have insurance that covers all or part of your prescription medication expenses?

هل لديك تأمين يغطي كل أو جزء من نفقات الأدوية الخاصة بك؟

- Yes- all expenses / نعم - جميع النفقات
- Yes- part of my expenses / نعم - جزء من النفقات
- No / لا
- I don’t know / لا أعرف

164. How satisfied are you with the healthcare services you receive?

ما مدى رضاك عن خدمات الرعاية الصحية التي تتلقاها؟

- Very satisfied/ راض جداً
- Satisfied/ راضٍ
- Neither satisfied or unsatisfied/ لا راضي ولا غير راضي
- Unsatisfied/ غير راضي
- Very unsatisfied/ غير راضي بشدة

165. To what extent do you trust healthcare professionals in Canada?

ما هو مدى ثقتك في أخصائي الرعاية الصحية في كندا؟

- Not at all ليس البتة /
- A little قليلاً /
- A moderate amount الى حد متوسط /
- Very much كثيراً جداً /
- An extreme amount الى ابعد الحدود /

166. How often do you require an interpreter present when accessing healthcare services in Canada?

ما هو مدى احتياجك إلى مترجم عندما تستخدم اي من الخدمات الرعاية الصحية في كندا؟

- Always/ دائماً
- Sometimes/ أحياناً
- Never/ أبداً

167. When needed, how often were you offered an interpreter?

عند الحاجة، كم مرة عُرِضَ عليك مترجمًا؟

- Always/ دائماً
- Sometimes/ أحياناً
- Never/ أبداً

168. Did you take the flu shot last year in Canada?

هل أخذت لقاح الأنفلونزا السنة الماضية في كندا؟

- Yes / نعم
- No / لا
- I don’t know / لا أعرف
- Prefer not to answer/ أفضل عدم الإجابة

# 169. Oral Health Cluster:

170. In general, how would you rate the health of your teeth and mouth?

بصورة عامة، كيف تقيم صحة فمك و أسنانك؟

- Excellent/ ممتاز
- Good/ جيد
- Fair/ مقبول
- Poor/ ضعيف
- Very poor/ ضعيف جداً

171. When was the last time you went to a dentist?

متى كانت آخر مرة زرت بها طبيب الأسنان؟

- Less than a year ago/ منذ أقل من سنة
- 1 year to less than 2 years ago / منذ أكثر من سنة و لكن أقل من سنتين
- 2 years to less than 3 years ago / منذ أكثر من سنتين و لكن أقل من ٣ سنين
- 3 years to less than 4 years ago / منذ أكثر من ٣ سنين و لكن أقل من ٤ سنين
- 4 years to less than 5 years ago / منذ أكثر من ٤ سنين و لكن أقل من ٥ سنين
- 5 or more years ago / منذ ٥ سنين أو أكثر
- Never / لم أزر طبيب الأسنان

172. How often do you usually visit the dentist?

كم مرة عادة تزور عيادة طبيب الأسنان؟

- More than once a year for checkups / أكثر من مرة كل السنة للفحص
- About once a year for checkups / مرة كل السنة للفحص
- Less than once a year for checkups / أقل من مرة كل السنة للفحص
- Only for emergency care / للطوارئ فقط

173. Do you have insurance that covers all or part of your dental expenses?

هل لديك تأمين يغطي كل أو جزء من نفقات العناية بالأسنان الخاصة بك؟

- Yes- all expenses / نعم - جميع النفقات
- Yes- part of my expenses / نعم - جزء من النفقات
- No / لا
- I don’t know / لا أعرف

Final Questions Cluster:

الاسئلة النهائية:

174. What is your favorite thing about Canada?

ما هو الشيء المفضل لك في/عن كندا؟

1. Child Information Cluster:

**معلومات الطفل:**

For this section, please answer about your eldest child who is younger than 18 years of age.

في هذا الجزء ، يرجى الإجابة عن طفلك الأكبر الذي عمره أقل من ١٨ سنة.

2. Participant ID:
3. Family ID:
4. Is the participant the mother or the father?

هل المشارك الأم او الاب؟

- Mother الأم /
- Father الاب /

5. How old is your child?

كم عمر طفلك؟

6. What is their gender?

ما هو جنسهم؟

- Male / ذكر
- Female / انثى
- Other / غير

7. In general, would you say your child’s physical health is:

بشكل عام ، هل تقول أن صحة طفلك الجسدية هي:

- Excellent/ ممتازة
- Very good/ جيدة جداً
- Good/ جيدة
- Fair/ مقبولة
- Poor/ سيئة

8. In general, would you say your child’s mental health is:

بشكل عام ، هل تقول أن صحة طفلك النفسية هي:

- Excellent/ ممتازة
- Very good/ جيدة جداً
- Good/ جيدة
- Fair/ مقبولة
- Poor/ سيئة

9. Please rate your child’s ability to communicate in English.

يرجى تقييم قدرة طفلك على التواصل باللغة الإنجليزية.

- Excellent/ ممتازة
- Very good/ جيدة جداً
- Good/ جيدة
- Fair/ مقبولة
- Poor/ سيئة

10. Please rate your child’s ability to communicate in your native language.

يرجى تقييم قدرة طفلك على التواصل بلغتك الأم.

- Excellent/ ممتازة
- Very good/ جيدة جداً
- Good/ جيدة
- Fair/ مقبولة
- Poor/ سيئة

11. What grade are they in in school?

في أي صف هم في المدرسة؟

- Grade 1 / الصف الاول
- Grade 2 / الصف الثاني
- Grade 3 / الصف الثالث
- Grade 4 / الصف الرابع
- Grade 5 / الصف الخامس
- Grade 6 / الصف السادس
- Less than grade 1 / أقل من الصف الاول
- Grade 7 / الصف السابع
- Grade 8 / الصف الثامن
- Grade 9 / الصف التاسع
- Grade 10 / الصف العاشر
- Grade 11 / الصف الحادي عشر
- Grade 12 / الصف الثاني عشر
- Grade 13 / الصف الثالث عشر
- Does not apply / ينطبق لا

12. Please rate your child’s performance in school.

يرجى تقييم أداء طفلك في المدرسة.

- Excellent/ ممتازة
- Very good/ جيدة جداً
- Good/ جيدة
- Fair/ مقبولة
- Poor/ سيئة
- Does not apply / ينطبق لا

13. How often does your child face discrimination in their school in Canada?

ما مدى مواجه طفلك التمييز في المدرسة في كندا؟

- Never ابداً /
- Seldom نادراً /
- Quite often احياناً /
- Very often في معظم الأحيان /
- Always دائماً /
- Does not apply / ينطبق لا

14. In general, to what extent do you think your child has experienced bullying from their peers in their school in Canada?

بشكل عام إلى أي مدى تعتقد أن طفلك قد تعرض للتنمر من أصحابه في مدرستهم في كندا؟

- Not at all ليس البتة /
- A little قليلاً /
- A moderate amount الى حد متوسط /
- Very much كثيراً جداً /
- An extreme amount الى ابعد الحدود /
- Does not apply / ينطبق لا

15. In general, to what extent do you think your child has bullied their peer(s) in their school in Canada?

بشكل عام إلى أي مدى تعتقد أن طفلك تنمر على أصحابه في مدرستهم في كندا؟

- Not at all ليس البتة /
- A little قليلاً /
- A moderate amount الى حد متوسط /
- Very much كثيراً جداً /
- An extreme amount الى ابعد الحدود /
- Does not apply / ينطبق لا

16. In general, to what extent does your child struggle to establish close ties and friendships in school?

بشكل عام إلى أي مدى يعاني طفلك من إقامة علاقات وثيقة وصداقات في المدرسة؟

- Not at all ليس البتة /
- A little قليلاً /
- A moderate amount الى حد متوسط /
- Very much كثيراً جداً /
- An extreme amount الى ابعد الحدود /
- Does not apply / ينطبق لا
